# Alterations in the host transcriptome *in vitro* following Rift Valley fever virus infection

**DOI:** 10.1038/s41598-017-14800-3

**Published:** 2017-10-30

**Authors:** Chelsea Pinkham, Bibha Dahal, Cynthia L. de la Fuente, Nicole Bracci, Brett Beitzel, Michael Lindquist, Aura Garrison, Connie Schmaljohn, Gustavo Palacios, Aarthi Narayanan, Catherine E. Campbell, Kylene Kehn-Hall

**Affiliations:** 1 National Center for Biodefense and Infectious Diseases, School of Systems Biology, George Mason University, Manassas, VA 20110 USA; 2 United States Army Medical Research Institute of Infectious Disease (USAMRIID), Frederick, MD 21702 USA; 3 DCE Consulting, Vienna, VA 22180 USA

## Abstract

Rift Valley fever virus (RVFV) causes major outbreaks among livestock, characterized by “abortion storms” in which spontaneous abortion occurs in almost 100% of pregnant ruminants. Humans can also become infected with mild symptoms that can progress to more severe symptoms, such as hepatitis, encephalitis, and hemorrhagic fever. The goal of this study was to use RNA-sequencing (RNA-seq) to analyze the host transcriptome in response to RVFV infection. G2/M DNA damage checkpoint, ATM signaling, mitochondrial dysfunction, regulation of the antiviral response, and integrin-linked kinase (ILK) signaling were among the top altered canonical pathways with both the attenuated MP12 strain and the fully virulent ZH548 strain. Although several mRNA transcripts were highly upregulated, an increase at the protein level was not observed for the selected genes, which was at least partially due to the NSs dependent block in mRNA export. Inhibition of ILK signaling, which is involved in cell motility and cytoskeletal reorganization, resulted in reduced RVFV replication, indicating that this pathway is important for viral replication. Overall, this is the first global transcriptomic analysis of the human host response following RVFV infection, which could give insight into novel host responses that have not yet been explored.

## Introduction

In the past decade, there has been an emergence of arbovirus-related diseases. With the increase in cases of arboviruses such as Zika, chikungunya, and West Nile viruses, there has been an effort to uncover the mechanisms of pathogenesis for these viruses, as well as develop appropriate therapeutics^1^. In the United States, Rift Valley fever virus (RVFV) is a Select Agent and a Category A priority pathogen due to its potential to cause severe economic distress and major health issues, as it has in Africa and the Arabian Peninsula for many years^2^. Furthermore, there has been increasing evidence that the U.S. and Europe are vulnerable to RVFV transmission due to the widespread range of competent vectors^3,4^. The virus is endemic to sub-Saharan Africa and has since spread to regions of the Arabian Peninsula, as well as Madagascar^2^.

RVFV causes major outbreaks among livestock, characterized by “abortion storms” in which spontaneous abortion occurs in almost 100% of infected, pregnant ruminants^5^. High mortality is also common in young animals that are infected with RVFV^5^. In addition to livestock, humans can also become infected through mosquito bites or handling of infected tissue. Human symptoms include a mild, febrile illness but can progress to more severe symptoms, such as hepatitis, encephalitis, and hemorrhagic fever^5^. A recent study indicated there was an association of miscarriage with RVFV infection in women from Sudan, which suggests that these more severe symptoms seen in pregnant livestock could possibly occur in pregnant women^6^. RVFV mortality rates have varied widely depending on the outbreak, but have overall been predicted to be around 0.5–1% by the World Health Organization (WHO)^4^. However, an outbreak in Mauritania in 2015 demonstrated a higher mortality rate of 42%, in which the most common form of the infection was the hemorrhagic form^7^. Overall, increased mortality rates, along with increased vector plasticity, demonstrates that the threat of RVFV spread to new nations is discernible and could have serious implications. To this day, a suitable FDA-approved treatment or vaccine remains outstanding. It is imperative that we develop a better understanding of pathways essential for RVFV replication and pathogenesis to aid in the development of necessary therapeutics and vaccines, which are vital to preventing the spread of this disease.

“Omics” approaches (genomics, transcriptomics, proteomics, etc.) can be used to enhance the full picture of the host response to infectious diseases. Comprehensive measurement of the host transcriptome has been made possible by Next-generation sequencing, where RNA is converted to a cDNA library and then sequenced using high-throughput sequencing technologies^8^. The goal of this study was to use RNA-sequencing (RNA-seq) to analyze host transcriptome changes in response to RVFV infection. To date, very few studies on global transcriptomic analysis in *Phlebovirus-*infected cells have been performed. Benferhat *et al*. investigated the presence of RVFV NSs protein at promoter regions of the host genome^9^. This study revealed pathways that were not previously known to be targeted by RVFV, such as cell adhesion, axonal guidance, coagulation, and Wnt signaling. In addition, a global RNA-seq analysis of Schmallenburg virus (SBV), another member of the *Bunyavirales* order showed differential gene expression between SBV and SBV lacking the nonstructural protein NSs^10^. They found that although NSs is efficient in shutting down the host immune response, there are many antiviral genes that are still upregulated following infection, identifying potential new antiviral factors that may be important for restricting infection. In this study, human small airway epithelial cells (HSAECs) were infected with two RVFV strains, the attenuated MP12 strain and the fully virulent ZH548 strain, to mimic an infection that would occur via aerosol in humans. While arthropods are mainly responsible for the transmission of RVFV, humans can also become infected by the inhalation of aerosols generated from infected tissue^11–13^. Samples were taken at early (3 hours), mid (9 hours), and late (18 hours) time points post infection and compared to their respective mock samples. Our work is the first global transcriptomic analysis of the human host response following RVFV infection, yielding insight into novel host responses that have not yet been explored.

## Materials and Methods

### Cell Culture

Human small airway epithelial cells (HSAECs) were acquired from Cambrex Inc., Walkersville, MD and maintained in Ham’s F12 medium supplemented with 10% Fetal Bovine Serum (FBS), 1% L-glutamine, 1% penicillin/streptomycin, 1% nonessential amino acids, 1% sodium pyruvate, 0.001% of 55 mM β-mercaptoethanol. Vero cells (ATCC, CCL-81) were maintained in Dulbecco’s modified minimum essential medium (DMEM) supplemented with 10% fetal bovine serum (FBS), 1% L-glutamine, and 1% penicillin/streptomycin. All cell lines were maintained at 37 °C with 5% CO_2_.

### Viruses and infections

Recombinant (r) MP12 and ZH548 viruses were rescued and titered as previously described^14,15^. All experiments with RVFV ZH548 were performed under biosafety level 3 (BSL-3) conditions. All work involving select agents is registered with the Centers for Disease Control and Prevention and was conducted at George Mason University’s Biomedical Research Laboratory, which is registered in accordance with federal select agent regulations. The rMP12-ΔNSs virus used in these studies contains a deletion of the NSs open reading frame (ORF), while the rMP12-ΔNSs-Luc virus contains a *Renilla* luciferase reporter in the place of NSs as previously described^16^. For viral infections, HSAECs or Huh7 cells were plated in a 6-well plate (7.5 × 10^5^ cells per well) and left to incubate overnight. The next day, cells were infected with the appropriate virus at the specified multiplicity of infection (MOI). Cells were incubated for 1 hour at 37 °C and 5% CO_2_. Infectious media was removed, washed twice with phosphate buffered saline (PBS) without Ca^2+^ and Mg^2+^, and complete media was added. The cells were left to incubate and collected using the appropriate method for downstream applications at the specified time points.

### RNA Sequencing

HSAEC samples infected with RVFV MP12 or ZH548 were collected at 3, 9, and 18 hours post infection in TRIzol LS (Ambion, 10296010). RNA was extracted per the manufacturer’s instructions and quantified using the LabChipGX (Perkin Elmer). RNA libraries were created using the TruSeq Stranded Total RNA Library Prep kit using 750 ng of input total RNA according to manufacturer’s protocol. The library preparation was automated using the Sciclone Workstation (Perkin Elmer). Prior to sequencing, ribosomal RNA was removed to enrich for mRNA. The completed libraries were quality checked with the LabChipGX and pooled, and then the pools were quantified with qPCR. Sequencing was performed using the Illumina platform.

### RNA-Seq Analysis and Bioinformatics

The viral genomes were downloaded from the NCBI RefSeq database. The accession numbers used are DQ380206.1, DQ375407.1, and DQ380151.1 for RVFV ZH548 and DQ380154.1, DQ375404.1, and DQ380208 for RVFV MP12. Viral segments were converted into a multi-FASTA file. Bowtie2^17^ was used to align trimmed paired-end reads to the viral genomes. Counts of reads aligned were used to estimate viral quantity in the samples. BRB-DGE (NIH/NCI) was used to align high-quality reads to the expressed genome of humans. This tool is a wrapper for TopHat^18^, HTSeq^19^, and Bowtie2^17^. Samples were normalized and differentially expressed genes were calculated using the R package DeSeq2^20–22^. Aligned read counts were standardized using the estimateSizeFactors function to adjust for their relative library sizes by dividing the read counts for each gene by the relative overall read counts for each sample. This controls for samples that may have been sequenced to different depths. The variance for each gene was estimated using the estimateDispersions function which calculates the square of the coefficient of variation (CV) across replicates, added to the shot noise (an estimate of the accuracy of observed read as compared to true gene expression). The CV dominates the calculations for highly expressed genes, while shot noise dominates the calculations for genes with small expression values. Differentially expressed genes were calculated using the nbinomTest function of DeSeq. In general, RNASeq data are not normally distributed across their large dynamic range, and additionally have generally large variances in gene expression across replicates, differentially. For these reasons, the negative binomial distribution has been chosen to estimate the distribution of the data sets, and differential expression is calculated using the estimated dispersions calculations, size factors, and negative binomial distribution using a modified Fisher’s exact test^22,23^. The datasets were un-log transformed and the average fold change and standard deviations were calculated for the triplicate samples for each infected/mock set per time point. A t-test was also performed on the triplicate samples for each infected/mock set per time point. Fold changes and p-values were input into Qiagen’s Ingenuity Pathway Analysis (IPA) program. Analysis in IPA was performed using the Core Analysis function. Genes changed more than 1.5X between infected and mock samples and p-values less than 0.05 were used for downstream analysis. These core analyses were then used to identify pathways, functions, and changed transcripts.

### RT-qPCR

HSAECs and Huh7s were plated in a 6-well plate (7.5 × 10^5^ cells per well) and mock-infected or infected with RVFV MP12 or ZH548 at MOI 5. Cells were collected using TRIzol LS (Ambion, 10296010) and RNA was extracted using the Direct-zol™ RNA MiniPrep (Zymo Research, R2050) per manufacturer’s protocol. DNase treatment was performed in conjunction with the RNA extraction using DNase I (Life Technologies, AM2222). Following DNase treatment and extraction, RNA was reverse transcribed into cDNA using a High Capacity RNA-to-cDNA kit with 1–2 µg of RNA (Applied Biosystems, 4387406). Real-time quantitative PCR (RT-qPCR) was performed using the StepOnePlus™ Real-Time PCR System (Life Technologies). TaqMan Gene Expression Assays were used for *ifit1* (Hs01911452_s1), *ifit2* (Hs01922738_s1), *ifit3* (Hs01922752_s1), *rsad2* (Hs00369813_m1), *actn2* (Hs00153809_m1), *arhgef6* (Hs00374477_m1), *tmsb10* (Hs01005565_g1) and *myh3* (Hs01074230_m1). Fold changes were calculated relative to 18 S ribosomal RNA and normalized to mock samples using the ΔΔCt method.

### Cellular Fractionation

HSAECs were seeded at 1.2 × 10^7^ cells in a T-225 flask (~80–90% confluency) and left to incubate overnight. Cells were mock-infected or infected with rMP12 or rMP12-ΔNSs, a mutant lacking the nonstructural protein NSs, at MOI 5 for one hour. At 18 hours post infection, cells were pelleted and washed once with PBS. Once the wash was removed, pellets were resuspended in 300 µL of Buffer A [10 mM KCl, 10 mM MgCl2, 10 mM HEPES, 1 mM EDTA, 1 mM DTT, and EDTA-free complete protease inhibitor cocktail (Roche) with 0.5% NP-40] and incubated on ice for 10 minutes. Samples were subjected to centrifugation (5,000 rpm for 5 minutes) and the supernatant was saved as the cytoplasmic extract. Pellets were then washed once with Buffer A, and then twice with PBS to remove any remaining cytosolic fraction. The pellets were then resuspended in Buffer B (450 mM NaCl, 1.5 mM MgCl2, 20 mM HEPES, 0.5 mM EDTA, 1 mM DTT, and EDTA-free complete protease inhibitor cocktail) and incubated for 10 minutes on ice. Following incubation, lysates were clarified by centrifugation (13,000 rpm for 10 minutes) and the remaining supernatant was saved as the nuclear extract. Fractions were quantified using Bradford reagent and confirmed by western blot, probing for GAPDH (cytoplasmic) and Lamin A/C (nuclear).

### Treatments

Integrin-linked kinase inhibitor, Compound 22, was purchased from EMD Millipore (407331) and dissolved into 10 mM stocks using dimethyl sulfoxide (DMSO). Veros or HSAECs were pretreated with the indicated concentrations of Compound 22 or DMSO alone in a 96-well plate (12,000 cells per well) for one hour prior to infection. Treatment was removed, cells were infected for one hour, and complete media with no drug was added following infection.

### Western Blot

Protein lysates were collected and analyzed by western blot as previously described^24^. In brief, primary antibodies against GAPDH (Cell Signaling, 5174), Lamin A/C (Cell Signaling, 4777), p-Akt1 Ser 473 (Cell Signaling, 4060), total Akt1 (Cell Signaling, 4691), IFIT2 (Abcam - ab113112), Viperin (Cell Signaling -13996 S), Actinin 2 (Thermo Fisher Scientific - PA5-27863), Alpha-Pix (Cell Signaling -4753 S), RVFV Nucleoprotein (BEI Resources, NR-43188), or HRP-conjugated actin (Abcam, ab49900) were diluted in 3% milk solution per the manufacturer’s recommended dilutions followed by the addition of the appropriate secondary antibody. Cropped, representative images are shown in main figures, but uncropped originals can be found in the Supplemental Figure 8.

### Plaque Assay

HSAECs or Veros were seeded in a 96-well plate at 12,000 cells per well and allowed to incubate overnight at 37 °C and 5% CO_2_. Treatments and infections were performed as described above. Supernatants were collected at the indicated time points and stored at −80 °C until use. Viral titers were determined by plaque assay as previously described^15^.

### Statistics

Unless otherwise noted, all statistical analysis was calculated using an unpaired, two-tailed student t-test using Graphpad’s QuickCalcs software. All graphs contain the mean and standard deviations with an n = 3.

### Data Availability

The datasets generated during the current study will be made available in the Gene Expression Omnibus (GEO) repository.

## Results

### Analysis of differentially expressed genes following RVFV infection

Despite a rapid increase in RNA-seq studies, very few studies focus on host transcriptome changes in the context of an acute viral infection. To obtain a broad picture of the host response to infection with the attenuated and fully virulent strains of RVFV, RNA-seq was used to analyze differential gene expression at the mRNA level. The mRNA was isolated and purified from mock infected or RVFV infected (MP12 and ZH548) HSAECs at 3, 9, and 18 hours post infection (hpi) and converted into cDNA libraries for sequencing. Three biological replicates were processed for each condition; while viral kinetic analysis was also performed to ensure infectivity and similar replication rates between MP12 and ZH548 (Supplemental Fig. 1). High-throughput sequencing generated an average of 10 million reads per sample. Approximately 56% of the reads were mapped to the human genome and there were anywhere between 10,000 and 20,000 genes expressed per replicate at each time point (Supplemental Fig. 2).

It has been previously shown that the nonstructural protein NSs is a potent suppressor of gene expression, specifically counteracting the production of interferon-beta (IFNB1) by its presence at the promoter of this gene^25,26^. NSs has also been shown to suppress transcription on a global level^26^, which adds a level of complexity in interpretation of RNA-seq data. While we did observed a trend in fewer reads at later time points in both MP12 and ZH548 infected cells, the variability between replicates was high; therefore these differences did not reach statistical significance (Supplemental Fig. 2). IFNB1 was identified as being upregulated via RNA-seq in MP12 infected cells at all time points, but only at 9 and 18 hpi in ZH548 infected cells (Supplemental Fig. 3). However, IFNB1 expression upregulated was only observed via RT-qPCR at 18 hpi in both HSAEC and Huh7 liver cells, with a larger fold-change detected in HSAECs. Robust NSs filament formation was observed in both HSAEC and Huh7 cells at 18 hpi (Supplemental Fig. 1), indicating that the increase in IFNB1 detected at 18 hpi was not due to lack of NSs filaments. Traditional housekeeping genes were analyzed for changes in gene expression over time to evaluate the overall influence of RVFV on host transcription (Supplemental Fig. 4). Six of the eight genes analyzed had a decreased in gene expression at 9 or 18 hpi in either MP12 or ZH548 cells. These were β-Actin (ACTB), GAPDH, the catalytic alpha subunit of protein phosphatase 1 (PPP1CA), phosphoglycerate kinase 1 (PGK1) and TATA-Box binding protein (TBP). In contrast, beta-2-microglobulin (B2M) was slightly upregulated in MP12 and ZH548 infected cells at 18 hpi and glucuronidase beta (GUSB) was not altered after infection. These results are in general agreement with the role of NSs in inhibiting transcription, but indicate that not all genes are affected in this manner.

**Figure 1:**
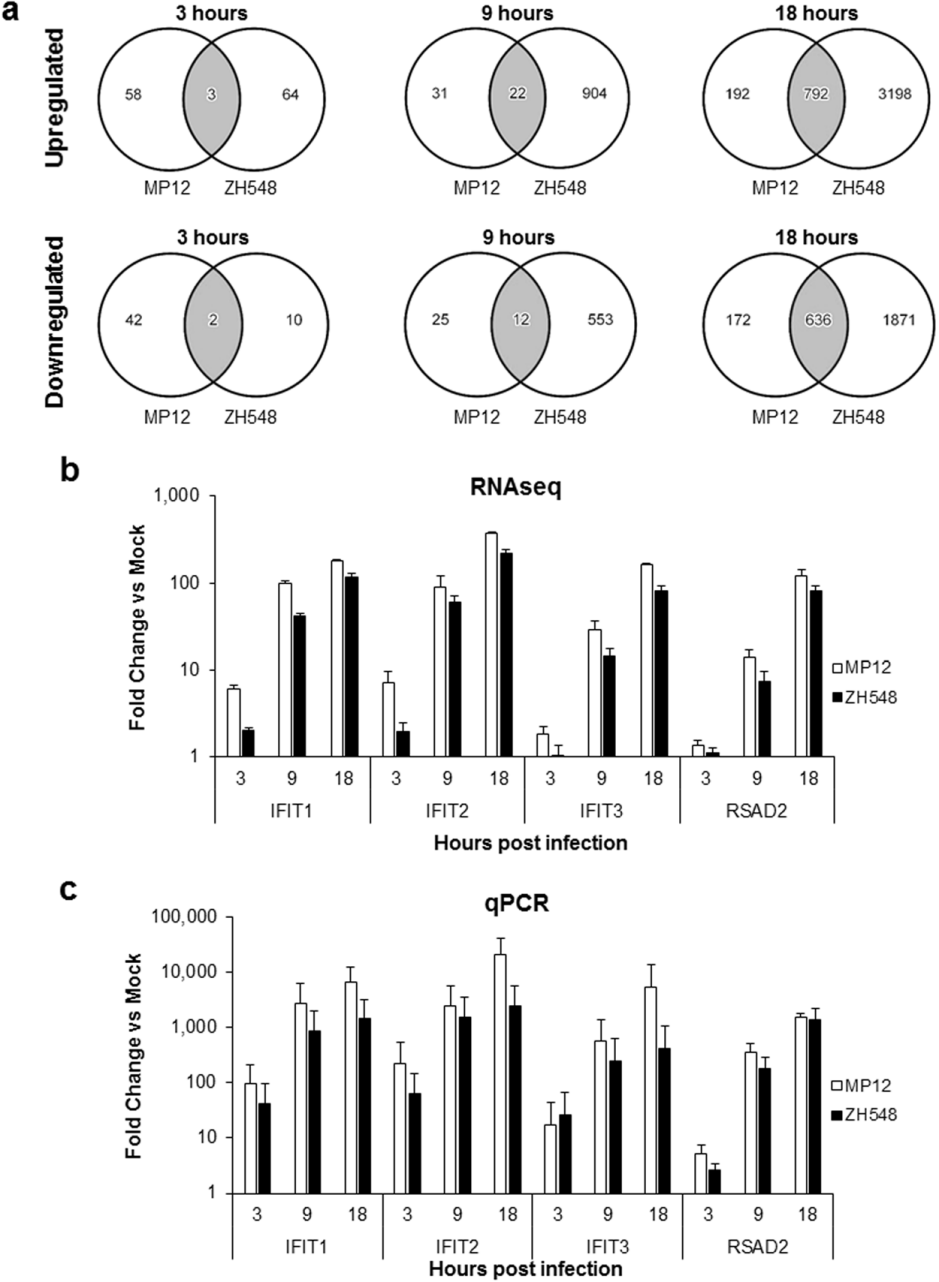
Analysis of differentially expressed genes in HSAECs following RVFV infection. (**a**) Venn diagrams depict the upregulated (top panel) and downregulated (bottom panel) genes changed in MP12 only (left), ZH548 only (right), or both (center, gray) at 3, 9, and 18 hours post infection. These genes were changed by 1.5-fold or more and had a p-value ≤ 0.05. (**b**) HSAECs were mock-infected or infected with MP12 or ZH548 at MOI 5 for one hour. Lysates were collected in Trizol LS, RNA was extracted and prepared for RNA sequencing. RNA-sequencing reads were normalized to the total reads, then fold changes were calculated against the uninfected, mock samples at the specified time point. (**c**) RT-qPCR confirmation of some of the top changed transcripts during all time points post infection. HSAECs were mock-infected or infected with MP12 or ZH548 at MOI 5 for one hour. Lysates were collected in Trizol LS, RNA was extracted using the Direct-zol™ RNA MiniPrep, and analyzed for RT-qPCR with TaqMan Gene Expression Assays against IFIT1, IFIT2, IFIT3, and RSAD2. Fold changes were calculated relative to 18 S ribosomal RNA and normalized to mock samples using the ΔΔCt method. Data are expressed as the Mean ± SD (n = 3).

Genes that had a change of 1.5-fold or more between mock and infected cells, and a p-value of 0.05 or less, were identified for each condition at each time point (Fig. 1a). At 3 hpi, minimal changes in gene expression were observed in MP12 or ZH548 infected cells with only five genes common between the two strains (Fig. 1a). At 9 hpi, there was a modest increase in differential gene expression in MP12 infected cells, while the number of upregulated genes sharply increased to 926 genes in ZH548 infected cells. Additionally, the number of downregulated genes at 9 hpi was 37 and 565 in MP12 and ZH548, respectively. At 18 hpi, there were over 3,000 upregulated genes total, with 192 that were unique to MP12 infected cells and 3,198 that were unique to ZH548 infected cells. Furthermore, there were over 2,000 downregulated genes at 18 hpi, with the most downregulated genes present in ZH548.

In a closer look at the individual genes, the most highly regulated genes were interferon-stimulated genes (ISGs) involved in the innate immune response. The most highly upregulated gene at 18 hpi in both strains was IFIT2, which was up 372-fold and 219-fold in MP12 and ZH548, respectively (Fig. 1b). Interferon-induced protein with tetratricopeptide repeats, or IFIT genes, are highly upregulated in response to type I and type III interferons^27^. Along with IFIT2, IFIT1 and IFIT3 were also increased following infection with both strains. Other ISGs that are increased upon infection are 2′-5′ oligoadenylate synthase (OAS) family members OASL and OAS2 (Table 1), which facilitate the degradation of viral RNA and activation of interferon, although recent studies have shown that OASL can act as a pro-viral factor as well^28^. Furthermore, there were additional ISGs that were upregulated that play various roles in the immune response, including viperin (RSAD2), IFNB1, HECT and RLD domain containing E3 ubiquitin protein ligase 5 (HERC5), and interferon-induced with helicase C domain 1 (IFIH1/MDA5). In addition to gene expression changes related to the immune response, there were also multiple genes upregulated that were involved in ion transport. Sodium voltage-gated channel alpha subunit 3 (SCN3A) and two solute carriers SLC24A2 and SLC1A3 were highly altered following infection, indicating that ion transport could be important in response to infection. Other upregulated genes were involved in G-protein coupled receptor signaling, axonal guidance, TGF-β signaling, and transcriptional regulation.

**Table 1:** Top genes up- and down-regulated and downregulated in RVFV infection at 18 hpi.

RVFV vs Mock Infected Upregulated Genes at 18 hours				
Genes	MP12		ZH548	
	Fold Change	p-value	Fold Change	p-value
IFIT2	372.074	0.0055	219.32	1.02E-04
OASL	326.309	0.0013	197.65	1.53E-05
FAP	204.708	0.0038	122.164	0.0009
IFIT1	179.623	2.24E-05	115.485	9.37E-05
IFIT3	160.983	0.0025	80.641	0.0003
IFIH1	128.079	0.0008	75.775	0.0003
RSAD2	120.164	0.0015	81.874	0.0001
SCN3A	99.068	0.008	51.502	0.0004
ARHGAP15	82.373	0.0145	47.047	2.26E-06
SEMA3D	79.017	0.0112	40.6	0.0013
SLC24A2	73.442	0.0037	51.379	3.80E-05
ACVR1C	72.267	0.0142	55.104	0.0001
USP44	72.13	0.0056	45.513	0.0011
MGAM	70.02	0.0028	49.287	6.60E-06
ATF3	60.124	0.0059	44.916	1.90E-07
KLF4	56.562	0.0153	57.2	0.0069
OAS2	49.92	6.61E-07	26.37	0.0001
SLC1A3	49.884	0.0355	23.324	0.0002
HERC5	37.663	0.0062	25.119	4.30E-05
IFNB1	37.58	0.0034	26.035	6.40E-05
**RVFV vs Mock Infected Downregulated Genes at 18 hours**				
	**MP12**		**ZH548**	
**Genes**	**Fold Change**	**p-value**	**Fold Change**	**p-value**
HIST1H1D	−12.664	0.088	−6.987	0.023
ID3	−9.412	0.202	−6.443	0.046
STC2	−7.652	0.024	−5.967	0.138
MED22	−7.053	0.044	−2.591	0.07
HIST1H2BD	−6.99	0.09	−5.949	0.023
CDCA4	−6.29	0.007	−2.861	0.052
PIM1	−6.204	0.034	−4.776	0.004
CBX8	−5.735	0.031	−3.7	0.047
ARL4C	−5.701	0.001	−4.263	0.001
TMSB10	−5.696	0.119	−6.203	0.003
TMEM60	−5.604	0.02	−4.543	0.001
NXT1	−5.264	0.048	−4.954	1.62E-04
POP7	−5.153	0.049	−1.679	0.21
HILPDA	−5.013	0.009	−5.401	2.09E-04
SLC38A2	−4.47	0.012	−5.888	0.004
MIR17HG	−4.174	0.006	−7.387	0.001
MSH6	−4.078	0.015	−6.019	0.001
CKS2	−3.987	0.001	−5.607	0.001
LSM11	−3.966	0.008	−8.238	0.001
HYLS1	−3.665	2.09E-04	−5.914	2.74E-04

A few highly upregulated genes, IFIT1, IFIT2, IFIT3 and RSAD2, were chosen for confirmation by RT-qPCR. When compared to the mock samples, these genes were significantly upregulated by more than 10-fold by 9 hpi in both strains per RNA-seq analysis. RT-qPCR analysis demonstrated the same trend in gene upregulation, with even greater changes observed (Fig. 1c). These results reveal that the data uncovered through RNA-seq could be confirmed through classical RT-qPCR analysis.

Genes that were downregulated in response to infection included a much more diverse set of genes with various roles throughout the cell (Table 1). Multiple genes were involved in chromatin structure and remodeling, including chromobox 8 (CBX8) and histone cluster 1 H1 family members (HIST1H1D and HIST1H2BD). In addition, genes involved in transcriptional regulation were highly downregulated, including cell division cycle associated 4 (CDCA4), inhibitor of differentiation 3 (ID3), and mediator complex subunit 22 (MED22). Nuclear transport factor 2-like export factor 1 (NXT1) was downregulated, which functions as a nuclear export factor and stimulates export of tRNA and mRNA^29^. Pre-mRNA processing was another function represented in the pool of downregulated genes, including LSM11 U7 small nuclear RNA associated (LSM11) and POP7 homolog (POP7), which are involved in histone 3′-end pre-mRNA processing and the generation of mature tRNA molecules, respectively. Furthermore, the host gene for miR-17-92 cluster was shown to be decreased following infection, which was also found independently by our group in a previous miRNA analysis study (unpublished data). While other solute carrier family genes were upregulated following infection, solute carrier family 38 member 2 (SLC38A2) was downregulated following infection in both strains. SLC38A2, also known as SNAT2 and ATA2, has been shown *in vivo* to play a role in the transportation of maternal nutrients to the fetus through the placenta^30^. Overall, these data indicate that there are a wide variety of genes altered following infection. Many of these genes would be of great interest for further investigation into their role in RVFV infection.

### Top canonical pathways that are transcriptionally altered following RVFV infection

**Figure 2:**
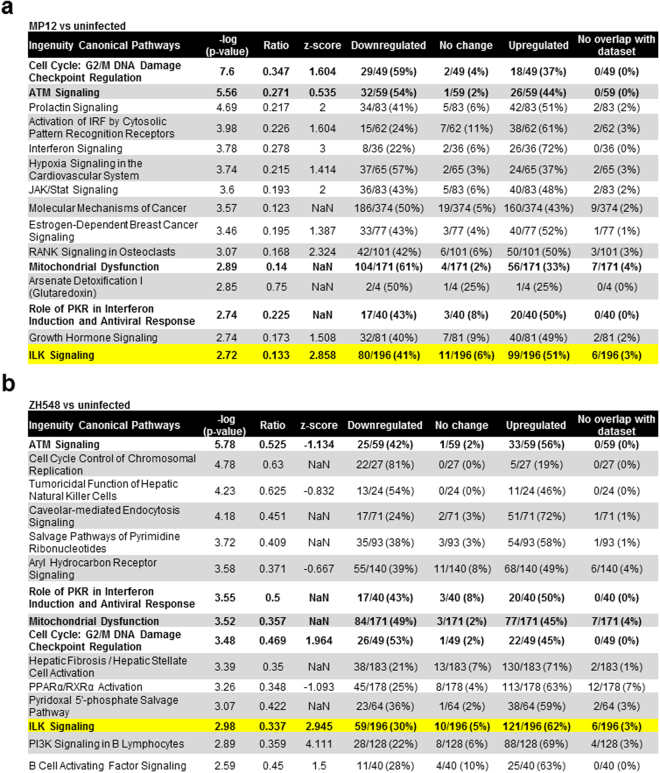
Top canonical pathways that are transcriptionally altered following RVFV infection. The top 15 canonical pathways changed in MP12 (**a**) or ZH548 (**b**) infection at 18 hours post infection. Depicted is the canonical pathway, p-value of the right-tailed Fisher’s exact test, ratio (how many genes in the changed in the pathway over the total number of genes in the pathway), z-score (predicted directionality; NaN = not a number, unable to predict directionality), and the number of genes in each category (upregulated, downregulated, no change, or no overlap with dataset). Pathways highlighted in bold are pathways that were common among the two strains for the top 15 pathways. The p-value is represented as the -log(p-value), which is the result of the Fisher’s exact test, and in this case, the larger the value, the more significant. The ratio is the percentage of pathway coverage, however this can be biased due to differing pathway sizes. The z-score is the predicted directionality of the pathway, where a value of more than 2 or less than −2 is considered significant (NaN = not a number; unable to predict directionality). The final four columns are the number and percentage of genes that are upregulated, downregulated, had no change, or did not overlap with the dataset. Integrin-linked kinase (ILK) signaling is highlighted in yellow.

While RNA-seq can provide an abundance of information on specific genes and how they are altered, our goal was to identify pathways that are imperative to either the host response or efficient RVFV replication. To this end, Qiagen’s Ingenuity Pathway Analysis (IPA) was used to explore highly altered pathways that are important for RVFV replication. Figure 2 shows the most significantly altered pathways (top 15) following infection with MP12 (Fig. 2a) or ZH548 (Fig. 2b). Many of the pathways listed here were similar between the two virus strains, but did not make the cut off of the top 15 most significant canonical pathways for one of the strains. Among the top 15 altered canonical pathways for the MP12 strain, but not the fully virulent ZH548 stain, were those involved in regulation of the antiviral response, including activation of IRF by cytosolic pattern recognition receptor signaling and interferon signaling. Although it did not make the top 15 pathways in ZH548, these pathways were still upregulated following infection. This pathway has also been confirmed in previous studies, specifically showing the activation of interferon regulatory factor 3 (IRF-3), nuclear factor kappa-light-chain-enhancer of activated B cells (NF-kB), and activator protein 1 (AP-1) in ZH548-infected cells^26^. Other pathways were altered in both MP12 and ZH548 infection, including G2/M DNA damage checkpoint, ATM signaling, mitochondrial dysfunction, role of PKR in interferon induction and antiviral response, and ILK signaling. These pathways, apart from ILK signaling, have been previously shown by our group and others to be altered following RVFV infection, indicating the reliability of the data^24,31–37^. In ZH548, cavaeolar-mediated endocytosis was highly altered following infection, which is the primary way in which RVFV enters mammalian cells^38^. Therefore, these data reveal ILK signaling as a novel pathway upregulated following infection with both RVFV strains.

### Integrin-linked kinase (ILK) signaling pathway is altered following RVFV infection

**Figure 3:**
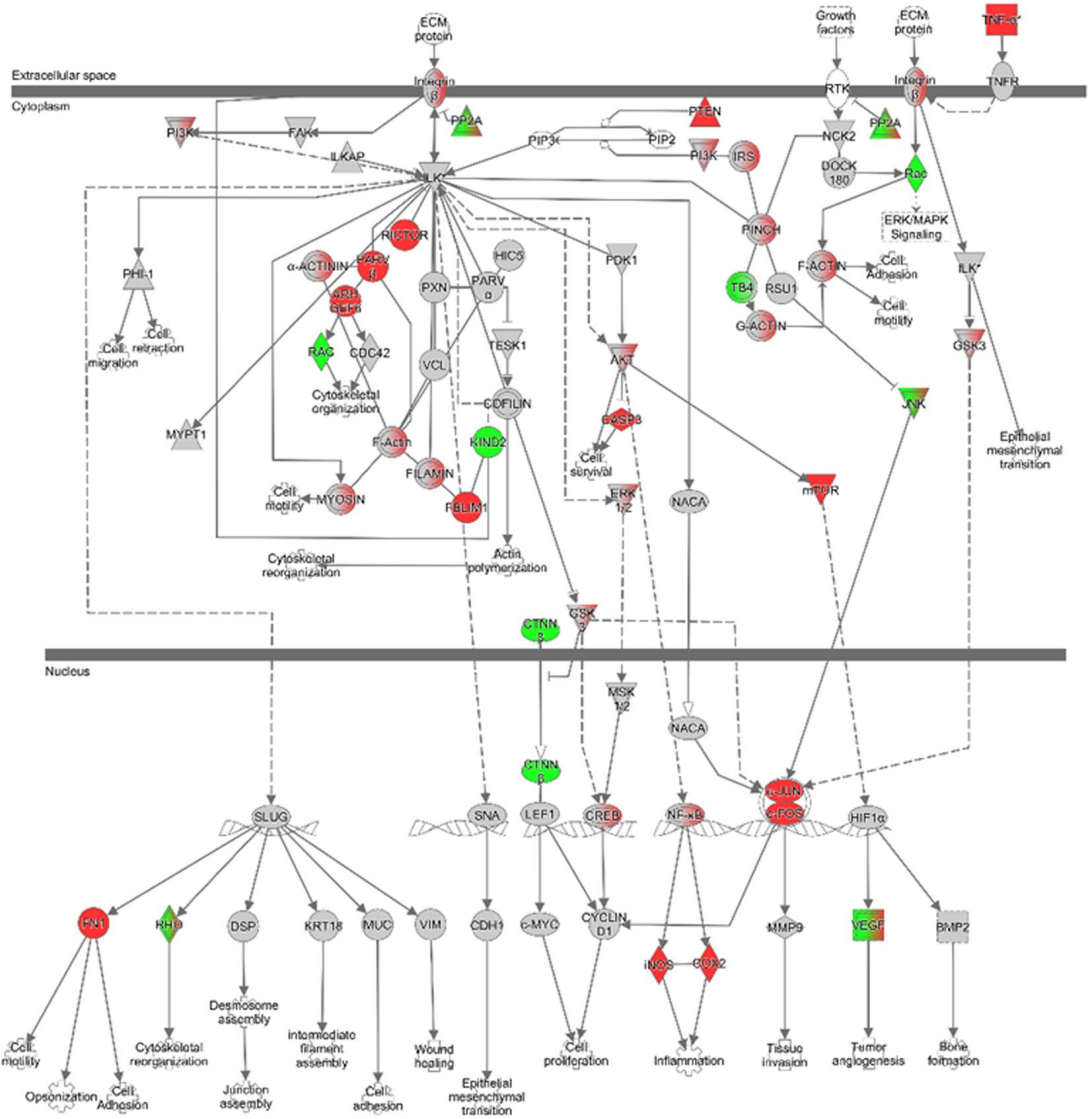
Integrin-linked kinase (ILK) signaling pathway is altered following RVFV infection. Changes in differential gene expression depicted over the ILK signaling canonical pathway generated by IPA. Red indicates genes that are upregulated, while green represents genes that are downregulated. Gray indicates genes that are in the dataset, but did not meet the fold-change or p-value threshold. Color gradients indicate that a subunit of that complex is upregulated or downregulated.

Integrin-linked kinase (ILK) signaling is responsible for connecting the extracellular matrix (ECM) to the actin cytoskeleton, contributing to important functions such as cell adhesion, survival, and proliferation^39^. Therefore, this pathway makes a rather enticing target for viral manipulation. The z-score, or the measure of correlation between relationship direction and gene expression, was significant in both strains at 2.858 and 2.945 for the MP12 and ZH548 strains, respectively (Fig. 2). The gene expression changes in ILK signaling for both strains were also significant as shown by the Fisher’s exact test. Additionally, more than half the genes in this pathway were upregulated for both strains, indicating that this pathway may be important for RVFV replication. The ILK signaling canonical pathway, involves a diverse set of players that participate in cell adhesion, cytoskeletal reorganization, and cell mobility. IPA allows for RNA-seq datasets to be placed as an overlay on canonical pathways, providing an interactive and large picture of how the pathway is altered under various experimental conditions (Fig. 3). At 18 hpi, the ZH548 strain shows a considerable upregulation (red) in genes such as β-Integrin subunits, RICTOR, ARHGEF6, α-Actinin-2, β-Parvin, PINCH, and multiple myosins. These genes play a role in cytoskeletal organization, which could potentially be essential to RVFV. Previous studies have shown that β3-integrins are critical for the entry of Hantaviruses, also a part of the *Bunyavirales* order, into epithelial cells and can play a role in lung pathogenesis^40^. Although RVFV isn’t known for its lung pathogenesis, these data are intriguing given that our work was done using lung epithelial cells. Furthermore, non-muscle myosin heavy chain IIA (NMMHC-IIA), upregulated in our study, was revealed to be an important factor in the entry of severe fever with thrombocytopenia syndrome virus (SFTSV), as siRNA knockdown of this protein led to significant decreases in infection^41^. NMMHC-IIA is also important for the function of human vascular endothelial cells and platelets, which may also affect its role in viral pathogenesis.

**Figure 4:**
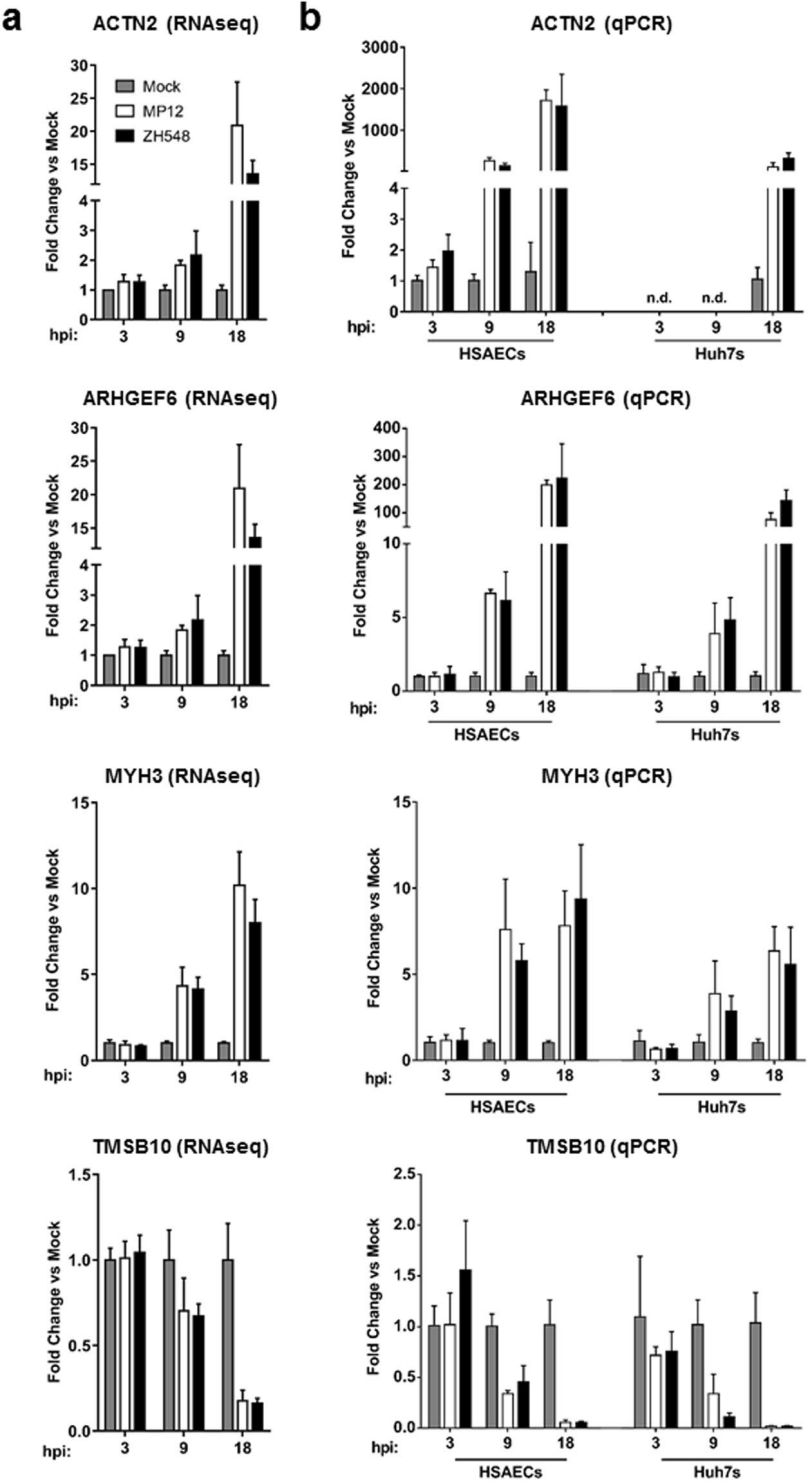
Real-time Quantitative PCR confirmation of altered ILK signaling genes. (**a**) RNA-sequencing reads were normalized to the total reads, then fold changes were calculated against the uninfected, mock samples at the specified time point. (**b**) RT-qPCR was performed to confirm changes seen in the RNA sequencing data. HSAECs or Huh7s were infected and RNA was collected in the same manner is Fig. 1C. Fold changes were calculated relative to 18 S ribosomal RNA and normalized to mock samples using the ΔΔCt method. Data are expressed as the Mean ± SD (n = 3).

To confirm significant changes in gene expression gathered from the RNA-seq data, RT-qPCR was performed. In this pathway, six genes were chosen for confirmation; α-actinin 2 (ACTN2), an actin cross-linking protein, Rac/Cdc42 Guanine Nucleotide Exchange Factor GEF 6 (also known as ARHGEF6, α-PIX, and cool-2), which plays a role in binding small GTPases and GDP/GTP exchange, myosin heavy chain 3 (MYH3), a major contractile protein, thymosin beta-10 (TMSB10), an inhibitor of actin polymerization, as well as key factors PINCH and Parvin-β, which act with ILK as a critical complex for transmitting signals between integrins and the actin cytoskeleton^42–45^. These genes were selected either due to being highly up- or down-regulated (ACTN2, ARHGEF6, MYH3 and TMSB10) or being of interest due to their well characterized interaction with ILK (PINCH and Parvin-β). By RNA-seq, ACTN2, ARHGEF6, and MYH3 expression showed little to no changes at 3 hpi in both strains, and increasing to only a few fold change by 9 hpi (Fig. 4a). At 18 hpi, these genes demonstrated a robust increase of 10- to 20-fold in both MP12 and ZH548. The trend seen in the RNA-seq data was confirmed in RT-qPCR experiments, although the changes seen were greater by PCR. ACTN2 revealed a time-dependent increase in gene expression, reaching almost 2,000-fold change by 18 hpi. Similarly, ARHGEF6 also demonstrated an increase but of only 200-fold by 18 hpi. Furthermore, comparable fold changes were seen between both RNA-seq and RT-qPCR for TMSB10. Small changes in gene expression by RNA-seq, as seen with PINCH, Parvin-β, and IFNB1, were more difficult to confirm by traditional RT-qPCR methods (Supplemental Figs 3 and 5). In addition to confirming these genes in HSAEC samples, the four confirmed genes were also tested in Huh7 liver cells (Supplemental Fig. 5). Liver pathogenesis is extremely common in RVFV infections; therefore we wanted to see if the genes in this pathway were also changed in these cells. Similar to the HSAEC samples, ACTN2, ARHGEF6, and MYH3 all demonstrated a time-dependent increase in gene expression, while TMSB10 decreased in gene expression over time. Overall, these results indicate that the RNA-seq data was generally reproducible by RT-qPCR, and confirm that the ILK signaling pathway is altered at the mRNA level following RVFV infection. Furthermore, these changes in gene expression were also confirmed in an additional cell type that is traditionally targeted during RVFV infection.

### RVFV-induced increases in host cellular mRNA is not reflected at the protein level due to a block in mRNA export

**Figure 5:**
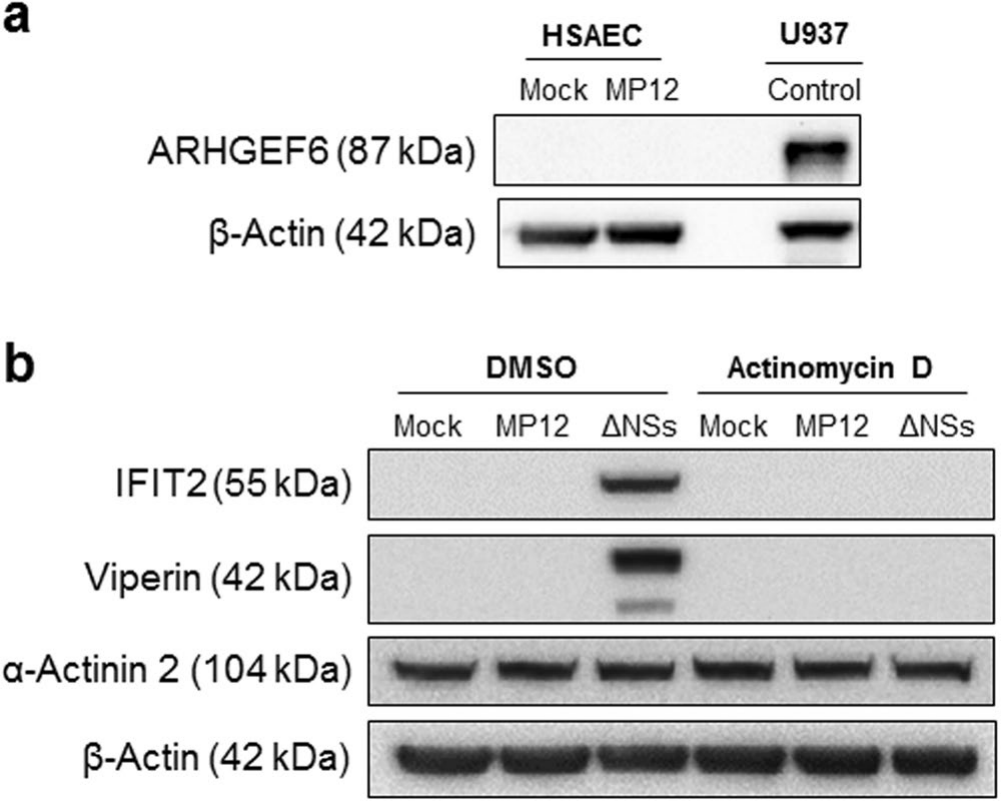
Increases in host cellular mRNA does not correlate with increases in protein expression. (**a**) HSAECs were mock-infected or infected with MP12 for one hour. At 18 hours post infection, cell lysates were collected and analyzed by western blot. As a control, U937 cell lysates were used. At 24 hours post infection, cell lysates were collected and analyzed in parallel by western blot. Membranes were probed for levels of ARHGEF6 (α-PIX) and β-Actin as a loading control. (**b**) HSAECs were mock-infected, infected with MP12, or infected with MP12-ΔNSs for one hour. Following infection, cells were treated with Actinomycin D (1 µg/mL) for the remaining time. At 18 hours post infection, cell lysates were collected and analyzed in parallel by western blot for levels of IFIT2, Viperin, α-Actinin-2, and β-Actin as a loading control. Cropped, representative images are shown here. Uncropped western blots can be found in Supplemental Fig. 8.

Following the confirmation of upregulated genes, the next step was to see if these changes were also reflected at the protein level. Protein expression of ARHGEF6 (α-PIX) was undetectable in HSAECs, both mock and RVFV MP12 infected (Fig. 5a). However, ARHGEF6 was highly expressed in U937 cells, which were used as a control. Further analysis of IFIT2, Viperin (the protein name for RSAD2), and α-Actinin-2 indicated no increase of protein expression in MP12-infected cells when compared to the mock (Fig. 5b). In contrast, α-Actinin-2 was abundantly expressed throughout all conditions, with no change in protein expression following infection with RVFV MP12 or in the presence of actinomycin D. These data indicate that the changes in mRNA expression correlate poorly with protein expression for the genes tested, therefore another mechanism between mRNA production and protein expression is playing a major role in RVFV-infected cells.

**Figure 6:**
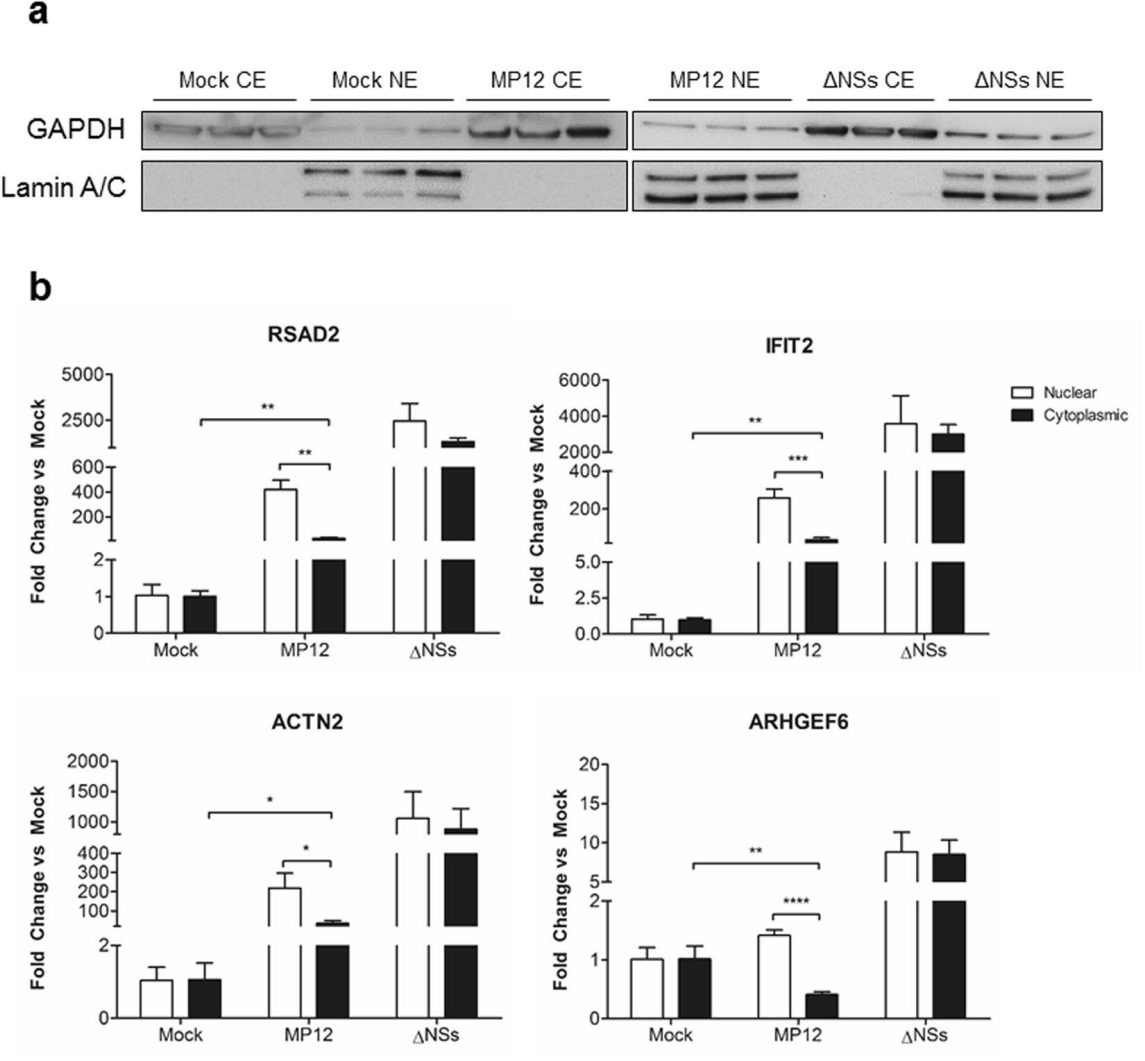
RVFV induces a block in host cellular mRNA export. (**a**) HSAECs were mock-infected, infected with MP12, or infected with MP12-ΔNSs for one hour. At 18 hours post infection, all samples were pelleted and cellular fractionation was performed in parallel. Equal amounts of protein were loaded and analyzed in parallel by western blot on two 10-well gels for successful fractionation. GAPDH was used at the cytosolic control, while Lamin A/C was used as the nuclear control. Cropped, representative images are shown here. Uncropped membranes can be found in Supplemental Fig. 8. (**b**) Following the cellular fractionation above, RNA was extracted from samples in parallel and analyzed by RT-qPCR. Analysis was done by normalizing to the 18 S rRNA and using the ΔΔCt method. Fold changes in the cytoplasmic and nuclear compartments were calculated by taking the fold change for that compartment over the total fold change of both compartments. Data are expressed as the Mean ± SD (n = 3). *p ≤ 0.05, **p ≤ 0.01, ***p ≤ 0.001, ****p ≤ 0.0001.

Copeland *et al*. previously showed that there is a defect in mRNA export in RVFV-infected cells, and this export defect correlates with NSs gene expression^46^. Their work demonstrated that host mRNA was localized to the nucleus of infected cells when compared to mock-infected cells, and that NSs is responsible for this phenomenon. An NSs dependent block in mRNA export could explain why there are many genes upregulated by RNA-seq, but of the genes tested here, there is no increase in protein expression. To this end, HSAECs were mock infected, infected with MP12, or infected with MP12-ΔNSs and nuclear and cytoplasmic fractionation was performed. Successful fractionation was confirmed by western blot analysis, probing for GAPDH (cytosolic fraction) and Lamin A/C (nuclear fraction). GAPDH was enriched in the cytosolic fraction, while Lamin A/C was only present in the nuclear fraction, indicating that the fractionation was achieved (Fig. 6a). RT-qPCR was performed on a combination of ISGs (IFIT2, RSAD2) as well as genes from the ILK signaling pathway (ACTN2 and ARHGEF6). In mock and MP12-ΔNSs infected cells, approximately 50% of each of the mRNAs was present in the cytoplasm (Fig. 6b). This corresponds to high protein expression of IFIT2 and Viperin in cells infected with the MP12-ΔNSs strain (Fig. 5b). Treatment with the transcriptional inhibitor, actinomycin D, decreased protein expression of IFIT2 and Viperin, indicating that the increases in protein expression were due to transcriptional activation. In contrast, MP12 infection resulted in a significant reduction in mRNAs in the cytoplasm (Fig. 6b). This was further confirmed by RNA fluorescence *in-situ* hybridization (FISH) targeting RSAD2, showing that RSAD2 mRNA is mainly nuclear following infection with MP12, but not with MP12-ΔNSs (Supplemental Fig. 6). In the case of RSAD2, IFIT2, and ACTN2, the mRNA in the cytoplasm of MP12-infected cells is increased over that of mock-infected cells. This may indicate that some mRNAs can get out of the nucleus, and that this host mRNA block is not a global phenomenon. These data confirm that there is an mRNA export block in MP12-infected cells, as shown here using individual genes.

### Inhibition of ILK signaling reduces RVFV replication

**Figure 7:**
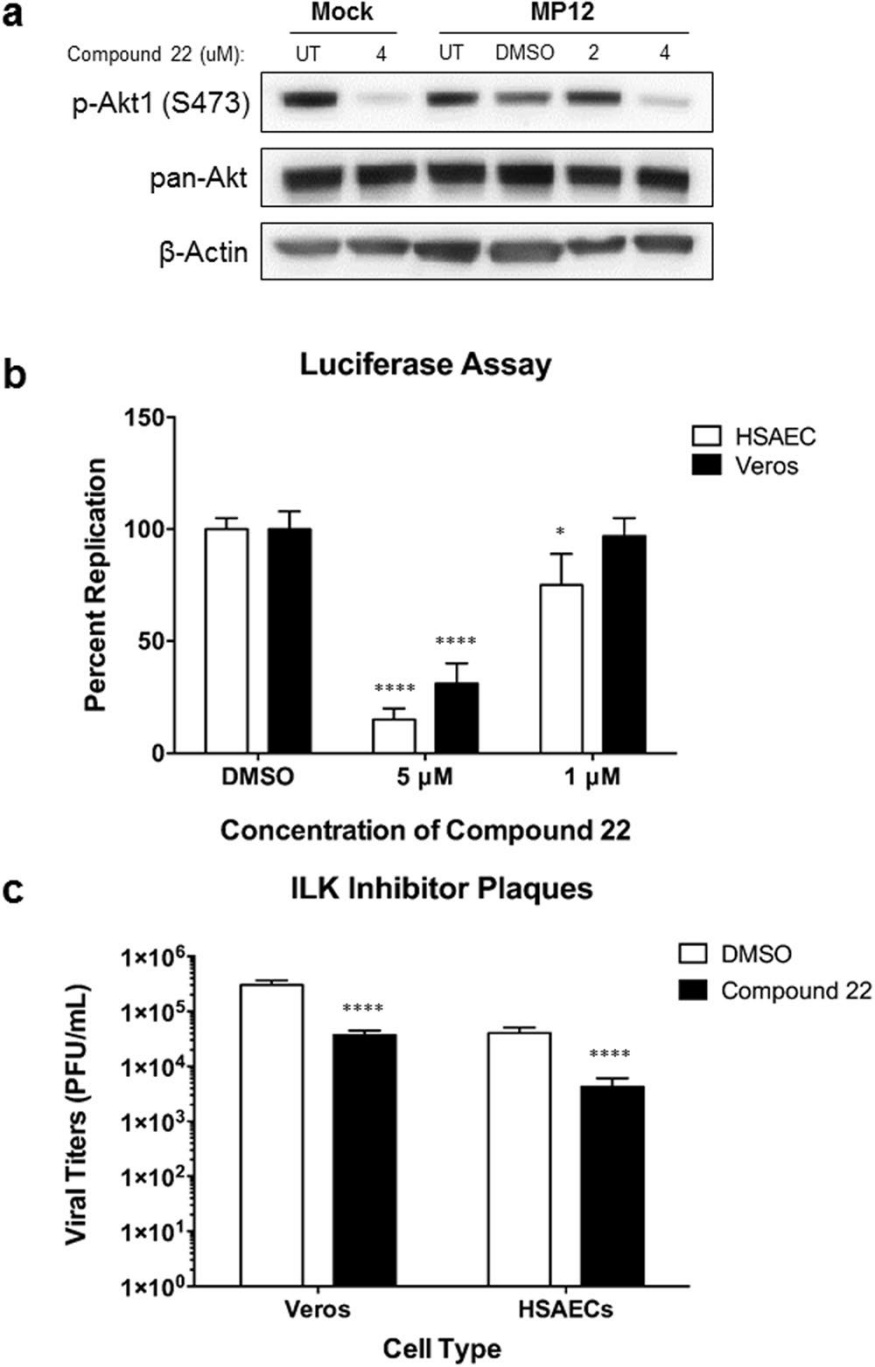
Pretreatment with Compound 22 reduces RVFV replication. (**a**) HSAECs were pretreated with 2 and 4 µM of Compound 22 or DMSO only in conditioned media for one hour. Following pretreatment, cells were infected with rMP12 at MOI 5 for one hour. Cells were washed once in PBS, then treated, conditioned media containing the inhibitor was replaced for the remaining time. At 24 hpi, cell culture media was removed and lysates were collected and analyzed by western blot. Membranes were probed for p-Akt (Ser 473), pan-Akt and β-Actin as a loading control. Cropped, representative images are shown here. Uncropped membranes can be found in Supplemental Fig. 8. (**b**) Vero cells and HSAECs were pretreated with Compound 22 (5 µM or 1 µM) or DMSO for one hour. Following treatment, cells were mock-infected or infected with MP12-Luciferase at MOI 0.1 for one hour. Cells were washed, then complete media was added. At 24 hpi, replication was measured by Promega’s *Renilla* Glo Luciferase assay according to manufacturer’s protocol. (**c**) Vero cells or HSAECs were pretreated and infected as in Panel B, then supernatants were collected at 24 hours post infection and analyzed by plaque assay. Data are expressed as the Mean ± SD (n = 3). *p ≤ 0.05; ****p ≤ 0.0001

ILK has been studied extensively in the context of various cancers and has emerged as a potential therapeutic target^47^. Although many studies have elucidated ILK and its role in various cancers, little is known about its role in the context of viral infections^48^. Given that ILK signaling was significantly upregulated at the mRNA level following RVFV infection, we used an ILK inhibitor, Compound 22, to examine the effects that blocking ILK activity has on viral replication. Compound 22 has been used to inhibit the phosphorylation of downstream targets, such as Akt, glycogen synthase kinase-3β (GSK-3β) and myosin light chain^49^. To confirm the ability of Compound 22 to inhibit ILK in our experimental system, alterations in the phosphorylation of a known downstream ILK target, Akt, was assessed. Cells were mock-treated or treated with DMSO, 2 µM, or 4 µM of Compound 22 for one hour, and then infected with MP12. Compound 22 treated media was added for the remainder of time following infection, and phosphorylation of Akt was analyzed by western blot. At 24 hpi, there was a considerable decrease in the phosphorylation of Akt (Ser 473) with 4 µM treatment with Compound 22, confirming that the inhibitor is inhibiting ILK activity in both mock- and MP12-infected cells (Fig. 7a). In addition, Compound 22 was also used to analyze whether ILK signaling had any effect on RVFV replication. Given the importance of the ILK pathway on cell motility and the cytoskeleton, we hypothesized that ILK signaling would be important for RVFV entry and/or early viral trafficking events. To test this hypothesis, a pre-treatment regimen, where Compound 22 was only present on the cells for one hour prior to infection, was used and the effects of Compound 22 on RVFV replication were measured by luciferase assay as well as plaque assay. For the luciferase assay, a recombinant virus was used in which the gene encoding the nonstructural protein NSs was replaced with the *Renilla* luciferase gene^16^. HSAECs or Vero cells pretreated with 5 µM of Compound 22 yielded a 70–80% decrease in viral replication in both cell types, while pretreatment with 1 µM resulted in little to no decrease in viral replication (Fig. 7b). Analysis by plaque assay revealed a reduction in viral titers by one log when cells were pretreated with Compound 22 (Fig. 7c). As a control, cell viability following Compound 22 treatment was measured using Cell Titer-Glo (Promega). No significant decrease in cell viability was detected at the 5 µM or 1 µM concentrations used for the viral inhibition assays (Supplemental Fig. 7). Overall, these data suggest that inhibition of ILK signaling results in a decrease in viral replication, indicating that ILK signaling is important for RVFV replication. Also, given that Compound 22 was only present on the cells for one hour prior to infection, this may indicate that ILK signaling used for entry or early events in the viral replication cycle.

## Discussion

Viral infections can initiate massive changes in the host transcriptome, disturb homeostasis to evade the host immune response, and create an environment more ideal for viral replication. In this study, our aim was to provide a broader picture of the host transcriptome in response to RVFV infection, as well as identify any commonalities or differences that may be important for pathogenesis. Here, we demonstrate changes to the host transcriptome following infection with the attenuated MP12 strain as well as the fully virulent ZH548. Among the top differentially regulated genes were ISGs, and these increases were confirmed independently by RT-qPCR. However, the induction of antiviral genes was not as robust in ZH548 infected cells as was observed in MP12 infected cells. For example, activation of IRF by cytosolic pattern recognition receptor signaling, interferon signaling, and Jak/Stat signaling were among the top 15 altered canonical pathways during MP12 infection, but not during ZH548 infection. The induction of IFIT1, IFIT2, IFIT3, and RSAD2 expression was also more robust in MP12 infected cells. These findings indicate that ZH548 infection results in a more dramatic inhibition of antiviral genes, which corresponds to its increased pathogenicity. Even given the high induction of ISGs, RVFV exploits multiple pathways to suppress the innate immune response, including the NSs-dependent suppression of the IFNB1 promoter, degradation of the general transcription factor TFIIH p62 through NSs interaction with FBXO3^50–52^, and NSs- mediated degradation of PKR via recruitment of the F-box proteins, FBXW11 and β-TRCP1^35–37,53,54^. Our RNA-seq analysis indicated upregulation of IFNB1in a time-dependent manner, but this increase could only be confirmed via RT-qPCR at 18 hpi consistent with literature indicating it is suppressed at earlier times of infection^26,55^.

In addition to the immune response, there were multiple genes upregulated that were involved in ion transport. Sodium voltage-gated channel alpha subunit 3 (SCN3A) and two solute carriers SLC24A2 and SLC1A3 were highly altered following infection. SLC24A2 is a solute carrier that is a potassium-dependent sodium/calcium exchanger. A previously published study showed that the modulation of potassium channels can inhibit Bunyamwera infection, which may indicate that the transport of these ions are important for RVFV infection^56^. Furthermore, SLC1A3, also known as GLAST, is responsible for sequestering excess glutamate and is important for maintaining low levels of extracellular glutamate. A recent study showed that this transporter was increased in astrocytes following Japanese encephalitis virus (JEV) infection, and suggested that it played a role in controlling the glutamate toxicity that is caused by neuronal damage following infection^57^. As for the importance of ion transport in RVFV-infected cells, further investigation is necessary to establish the mechanism. Another downregulated gene of interest was SLC38A2, also known as SNAT2 and ATA2, which helps transport nutrients across the placental barrier to the fetus. This could be particularly intriguing considering the characteristic abortion storms in pregnant ruminants, and the possible correlation of miscarriages in humans^6,58^. Overall, these genes are significantly altered in infected cells, and many of these genes could play a role in the phenotypes that are characteristic of RVFV infection.

One of the main virulence factors of RVFV is the nonstructural protein NSs. Its primary function is to dampen the host immune response, which is accomplished through multiple routes. NSs is the only viral protein that can gain access to the nucleus during RVFV infection, in which it forms characteristic filaments^59,60^. It was revealed that NSs binds with other co-factors to the promoter of IFNB1 to suppress transcription of this antiviral gene^61^. Furthermore, the characteristic filaments of NSs have been shown to sequester the p62 subunit of the TFIIH complex, resulting in a host transcriptional repression^61^. The large number of upregulated genes identified in our RNA-seq study was somewhat surprising, given the ability of NSs to suppress host transcription, particularly of the innate immune response genes. This indicates that NSs is not able to entirely block transcription of host antiviral genes in RVFV-infected cells, as many genes were significantly upregulated in infected cells. The ability of NSs to suppress host transcription may be a time-dependent phenomenon, as our data indicate very little change in gene expression at 3 hpi while the number of genes increases significantly at 18 hpi. It may be possible that RVFV can only repress host transcription in the early stages before the host immune response can overcome the block. Conversely, there is precedence in the literature for genes being upregulated following RVFV infection, even potentially dependent on NSs^9^. Previous work by the Bouloy and Bonnefoy groups found that genes within the coagulation cascade, which had NSs at their promoters, were upregulated at 8 hpi following RVFV infection. They speculated that NSs being present at these promoters may somehow provide an environment that is beneficial for cellular transcription.

To add another layer of complexity, a recent study demonstrated that there is a nuclear accumulation of mRNA following RVFV infection^46^. Cells infected with a virus lacking NSs did not show this phenotype, indicating that this phenomenon was dependent on NSs. This was recapitulated in our study, showing nuclear accumulation of specific mRNAs in RVFV-infected cells, but not cells infected with MP12-ΔNSs. Interestingly, this block does not seem to be exclusive, as some mRNAs are making their way into the cytoplasm of the cell, possibly for cap-snatching by the virus. One of the top downregulated genes identified through the RNA-seq study was NXT1, which functions as a nuclear export factor and stimulates export of tRNA and mRNA. This was significantly downregulated about 5-fold in both strains. A previous chromatin immunoprecipitation (ChIP)-chip study to analyze the presence of NSs at various promoter within the host genome revealed that NSs was present at the promoter of NXT1^9^. However, another study indicated that there were not any measurable results when looking at the effects of NSs on nuclear export pathway components, of which NXT1 was included^46^. Therefore, the mechanism by which NSs suppresses mRNA export is still unknown and is an important area for future research.

Analysis of downregulated genes through RNA-seq is further complicated due to RVFV performing cap-snatching^62,63^ and inducing the decay of mRNAs that possess a 5-terminal oligopyrimidine (5-TOP) motif in their 5′UTR^64^. Cap-snatching, stealing the 5′ end of cellular RNAs, to acts as a primer for viral transcription, results in cellular mRNA instability and ultimately degradation^65^. RVFV was found to selectively perform cap-snatching from mRNAs encoding cell cycle regulatory proteins^63^, which provides at least one mechanism by which RVFV induces cell cycle arrest^31^. During RVFV infection, the RNA decapping enzyme NUDT16 preferentially degrades 5′TOP mRNAs, especially those encoding translational protein machinery, resulting in host translational suppression^64^. RNA-seq analysis cannot differentiate between mRNAs that are downregulated at the transcriptional level or mRNAs that are degraded through a post-transcriptional decay mechanism. Therefore, genes and/or pathways that are identified here as being downregulated may be influenced through a transcriptional-independent mechanism.

Our study revealed alterations in many canonical pathways during RVFV infection, some of which have been previously shown by our group and others to be important for RVFV pathogenesis. Among the unique canonical pathways, ILK signaling was highly altered in both MP12 and ZH548 infected cells. ILK itself is not changed significantly at the mRNA level following RVFV infection. This is not too surprising given that kinases are often regulated at the protein level through modulation of localization, activity and/or binding partners. ILK is activated through interactions with the extracellular matrix and can go on to phosphorylate downstream effectors^66^. Alterations in ILK signaling were confirmed by RT-qPCR by selecting highly altered genes from the ILK signaling pathway. Viruses have long been known to alter the structure and function of the cytoskeleton in order to facilitate entry, transport, and egress^67,68^. Regulators of actin-based cytoskeletal reorganization were found to be transcriptionally upregulated at both S and G2 phases of the cell cycle^69^, which corresponds to the ability of RVFV to induce S-phase arrest^31^. While the actin cytoskeleton can act as an initial barrier to incoming viruses, it can also be manipulated to assist in production of virus factors, assembly, endocytosis, and cell-to-cell transmission^68^. Monomeric G-actin may play a role in replication and assembly of negative-stranded RNA viruses, where actin possibly stabilizes the binding of RNA-dependent RNA polymerase (RdRp) to the nucleocapsid, which encapsidates the viral RNA^70^. Additionally, a recent study done with Bunyamwera virus (BUNV) showed that multilamellar structures and extracellular actin filament bundles formed during egress, facilitating viral release and spread to neighboring cells^71^. They further hypothesized that these multilamellar structures and filament bundles could be important for cell attachment, facilitating viral spread without leading to detachment and death. Studies have indicated that ILK is present at focal adhesions and cell-to-cell adhesion sites, and can also be recruited directly or indirectly to integrin tails^72^. While ILK has been studied widely in cancer, its role in viral infections is poorly understood. Pretreatment with the ILK inhibitor Compound 22 resulted in a considerable decrease in RVFV replication by both luciferase assay and by plaque assay. Furthermore, these experiments were performed using only a one-hour pretreatment, indicating that Compound 22 may have some effect on entry or an early stage of the viral life cycle. We hypothesize that RVFV may be utilizing the ILK signaling pathway in order to facilitate actin cytoskeletal reorganization. While Compound 22 showed efficacy in decreasing RVFV replication, the mechanism by which ILK facilitates infection remains to be seen. Interestingly, a previous study indicated that ILK plays an important role in the trafficking of cavaeola to and from the cell surface, the primary way in which RVFV enters the cell^73^. Future work will encompass elucidating the specific role of ILK in RVFV infection.

In conclusion, these data represent the first overall transcriptomic analysis of RVFV-infected cells at multiple time points throughout infection. This dataset provides a plethora of information on how the host responds to RVFV on an mRNA level. While previous publications have indicated that NSs can cause global transcriptional suppression, it is clearly not able to inhibit transcription of all genes, even those that are antiviral. On the other hand, there are many challenges that arise when interpreting these data. The RVFV-induced suppression of transcription, as well as the possible block in mRNA export could be problematic. Many of these genes may be upregulated at the mRNA level, but may not be transported out of the nucleus to be translated into fully functioning proteins. Regardless of the mRNA export block, our study has demonstrated that these upregulated pathways can still be important for viral replication, as this study reveals a novel role for ILK signaling in RVFV infection. This overall transcriptomic analysis yields insight into the host response as well as into the genes responsible for RVFV pathogenesis.

## Electronic supplementary material

Supplemental Figures
