# Supplementary material for: Alterations in the host transcriptome in vitro following Rift Valley fever virus infection
Source: Sci Rep. 2017 Oct 30;7:14385. doi: 10.1038/s41598-017-14800-3 (PMC5662566; doi:10.1038/s41598-017-14800-3)
Supplement: Supplementary file 1 — Supplemental Figures [file 41598_2017_14800_MOESM1_ESM.pdf]

Supplemental Figures

Alterations in the host transcriptome *in vitro* following Rift Valley fever virus infection

Chelsea Pinkham[1], Bibha Dahal[1], Cynthia L. de la Fuente[1], Nicole Bracci[1], Brett Beitzel[2], Michael Lindquist[2], Aura Garrison[2], Connie Schmaljohn[2], Gustavo Palacios[2], Aarthi Narayanan[1], Catherine E. Campbell[3], Kylene Kehn-Hall[1*]

[1]National Center for Biodefense and Infectious Diseases, School of Systems Biology, George Mason University

[2] United States Army Medical Research Institute of Infectious Disease (USAMRIID)

[3]DCE Consulting

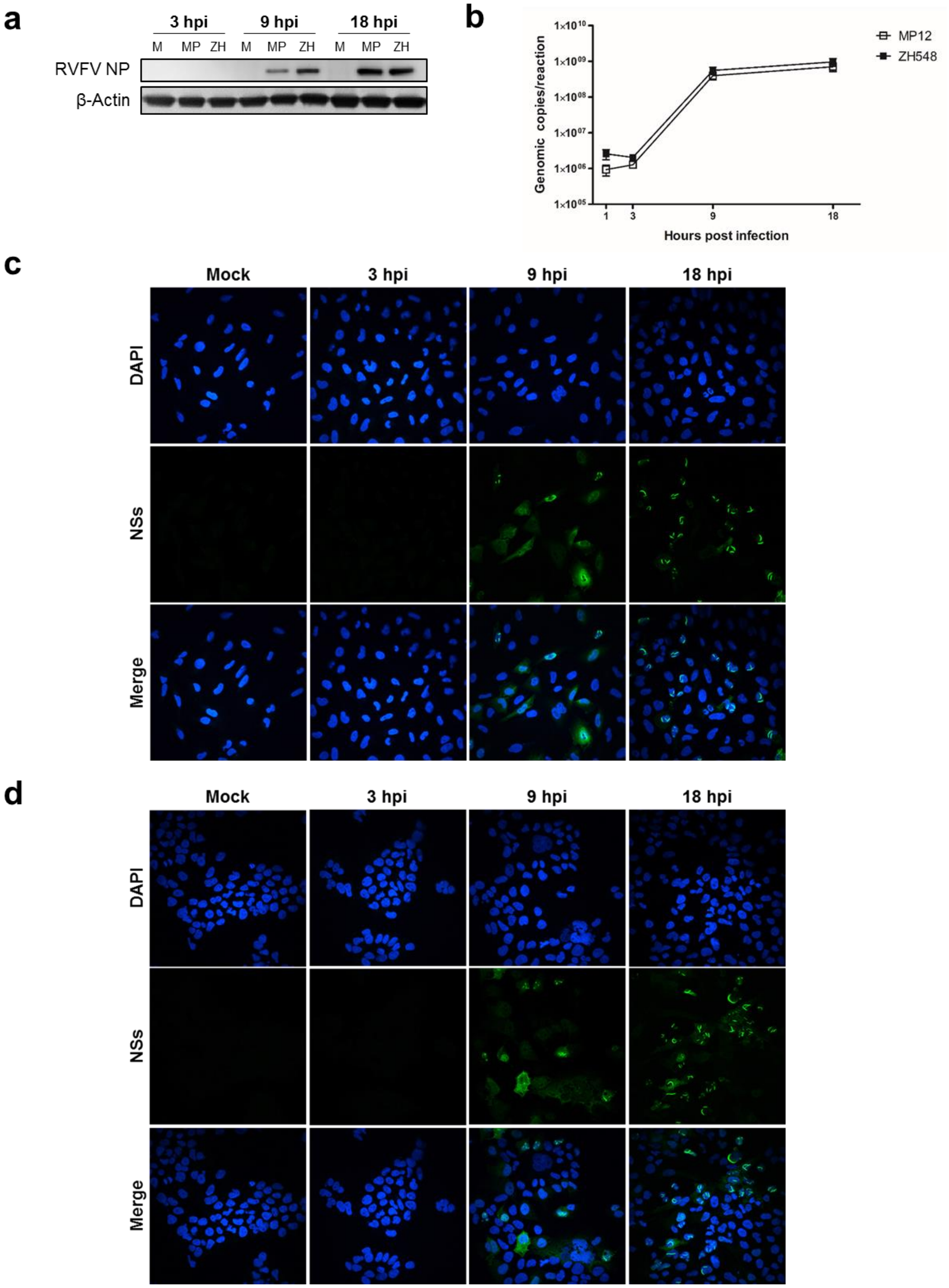

Supplemental Figure 1. RVFV MP12 and ZH548 replicate at similar rates. A) HSAECs were mock-infected (M) or infected with MP12 (MP) or ZH548 (ZH). At the indicated times post infection, cell lysates were collected and analyzed by western blot for RVFV nucleoprotein (RVFV NP) and β-Actin as a loading control. B) HSAECs were infected as stated in (A) and at the indicated time points, cells were lysed and RNA was extracted. RT-qPCR analysis was performed probing for the RVFV genome. Data is represented as the average genomic copies per reaction for three replicates. HSAECs (C) or Huh7s (D) were infected with MP12-Flag-NSs at MOI 5 for one hour, and fixed in 4% paraformaldehyde at the indicated time points. Cells were processed and probed for α-Flag antibody (NSs, green) and DAPI to stain the nuclei.

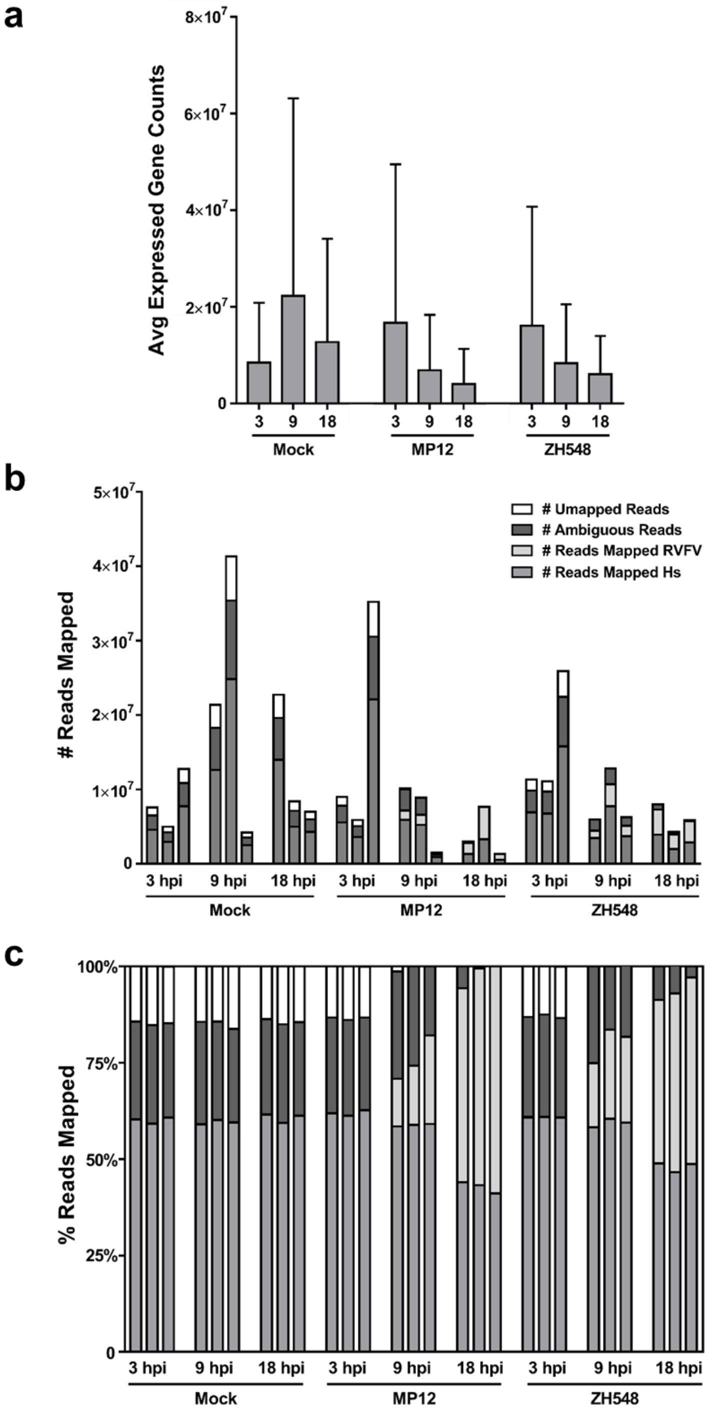

Supplemental Figure 2. RNA-seq reads mapped to the human and RVFV genomes. A) The average number of expressed genes for each time point. RNA-seq reads were trimmed and analyzed, and the raw number of reads (B) as well as the percent of the total reads (C) that were mapped back to the human genome (# Reads Mapped Hs) and the RVFV genome (# Reads Mapped RVFV) are displayed. The number and percent of ambiguous reads (reads that map to more than one location) as well as unmapped reads are also included.

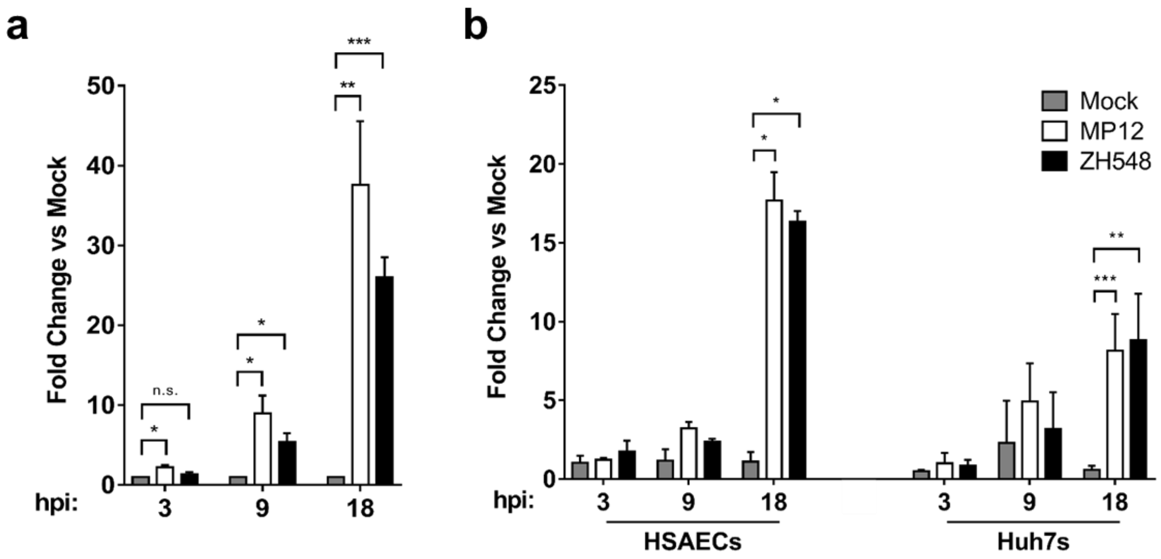

Supplemental Figure 3. mRNA analysis of interferon-β following RVFV infection. A) RNA-sequencing reads were normalized to the total reads, then fold changes were calculated against the uninfected, mock samples at the specified time point. B) RT-qPCR was performed to confirm changes seen in the RNA sequencing data. HSAECs or Huh7s were infected and RNA was collected in the same manner is Figure 1. Fold changes were calculated relative to 18S ribosomal RNA and normalized to mock samples using the ΔΔCt method. Data are expressed as the Mean ± SD (n = 3). * $p \leq 0.05$, ** $p \leq 0.001$, *** $p \leq 0.0001$

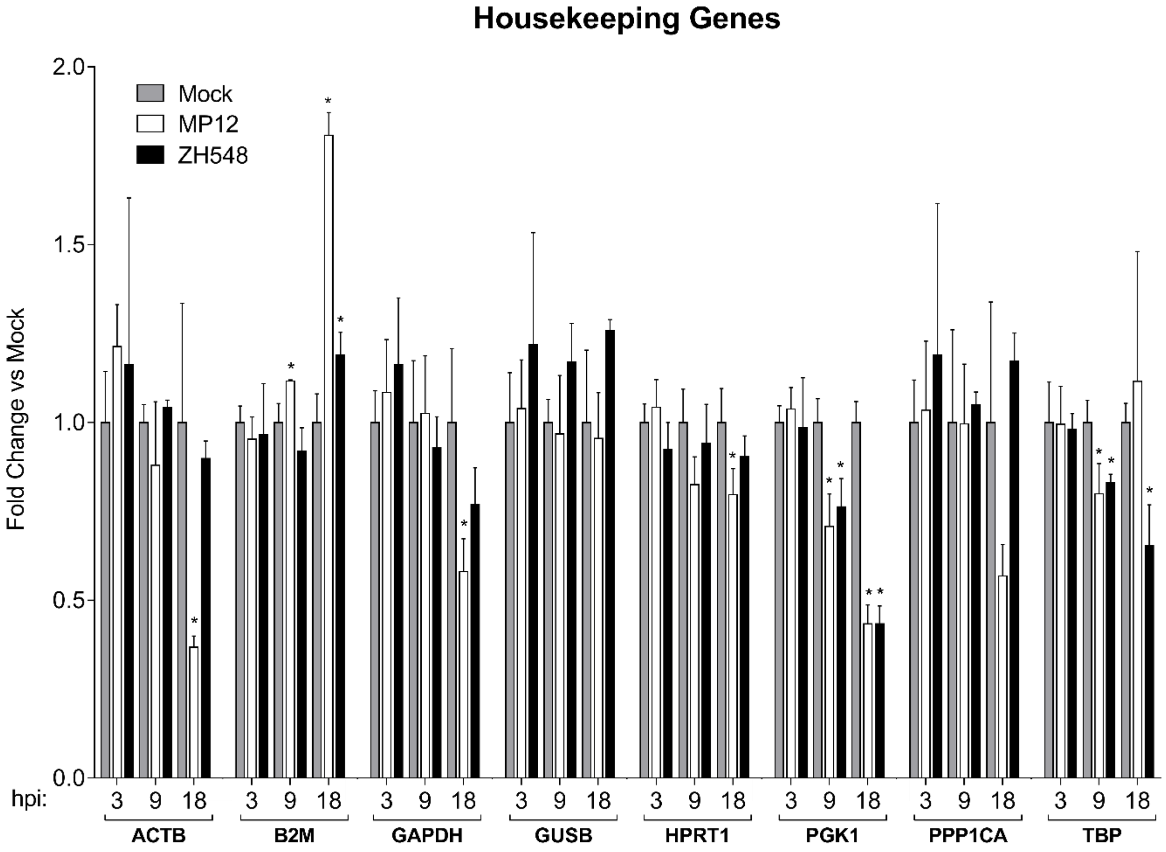

Supplemental Figure 4. Analysis of housekeeping genes over time through RNA-seq. Traditional housekeeping genes were pulled from the RNA-seq data and fold change was calculated compared to the average of the mock samples at the indicated time points. * $p \leq 0.05$

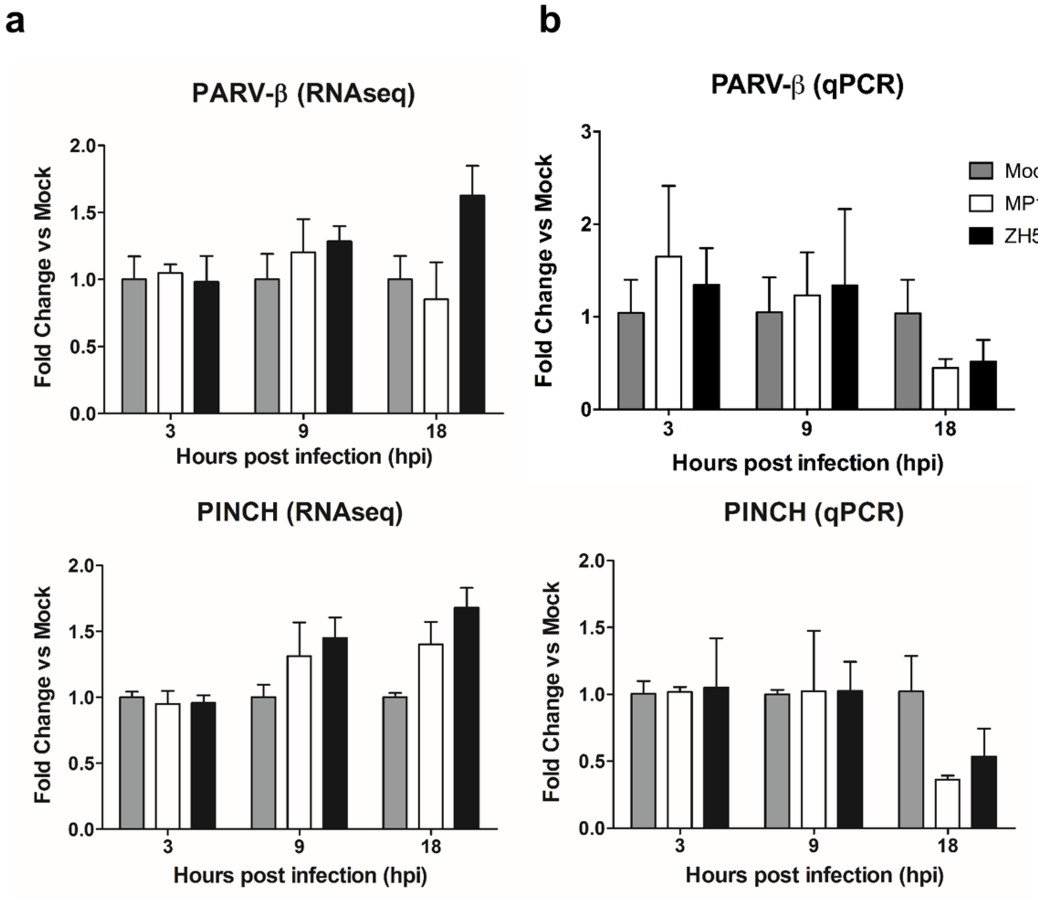

Supplemental Figure 5. Transcripts with lower fold changes were less likely to confirm by RT-qPCR. HSAECs were mock-infected or infected with MP12 or ZH548 and collected during the indicated time points. RNA was extracted and analyzed by RNA-seq (A) or RT-qPCR (B). Fold changes were calculated against the mock for RNA-seq. For RT-qPCR, the ΔΔCt method was used to calculated fold changes after being normalized to the 18S ribosomal RNA.

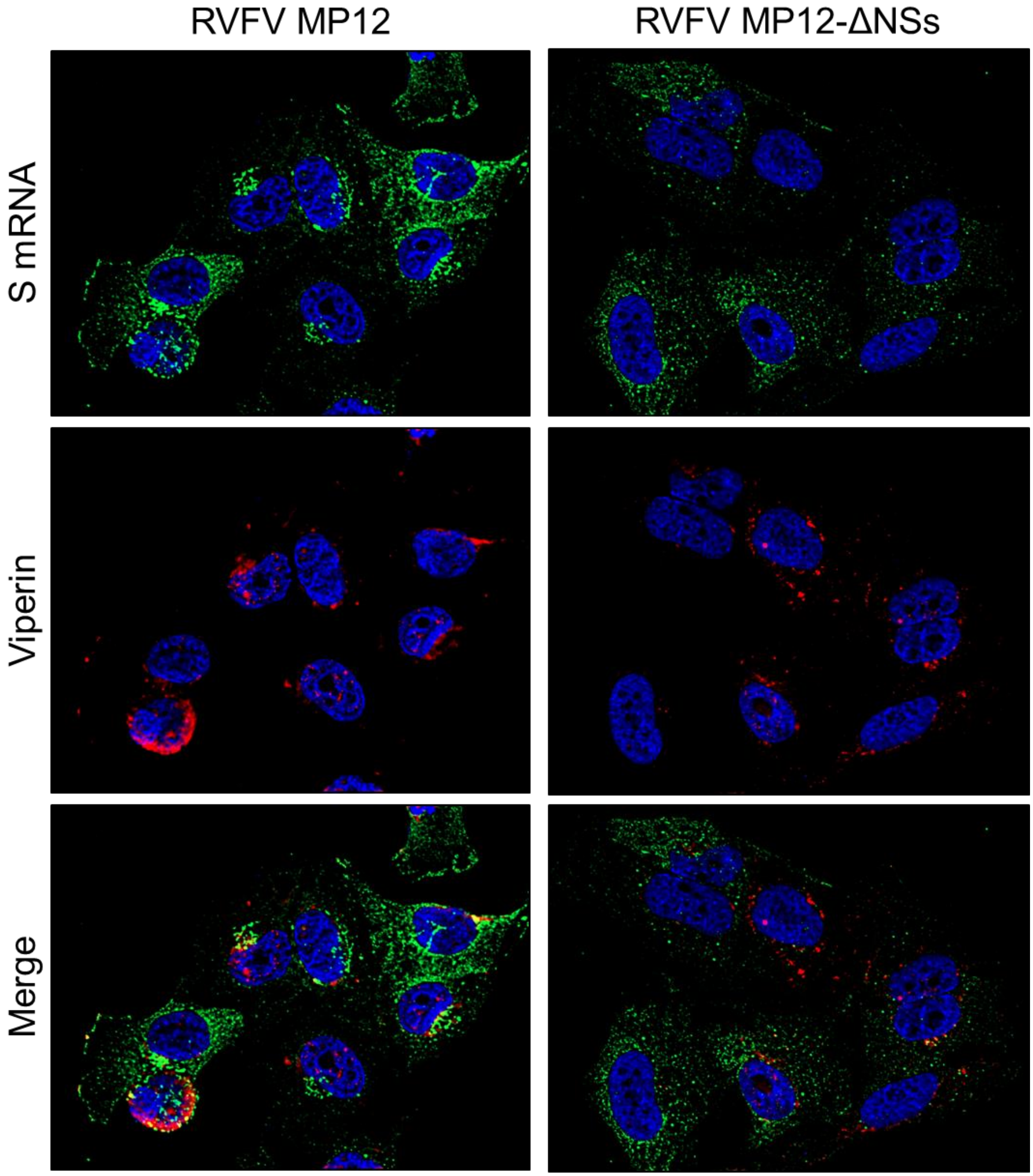

Supplemental Figure 6. RVFV alters the localization of RSAD2 (Viperin) mRNA following infection. HSAECs were grown on #1.0 coverslips and then infected with RVFV MP12 or RVFV MP12 ΔNSs. At 18 hpi, cells were then fixed in 3.7% formalin for 10 minutes, washed in PBS, and permeabilized in 70% ethanol at 4ºC for at least 1 hr. Cells were washed in formamide wash buffer and then incubated with 2.5 μM of the probe set in formamide hybridization buffer overnight at 37ºC overnight. The following day, samples were washed, rinsed in 2X SSC buffer, and then mounted. Coding sequences for RVFV MP12 S (accession number AF134530.1) and RSAD2 (Viperin) (AF442151.1) were obtained from GenBank.

a

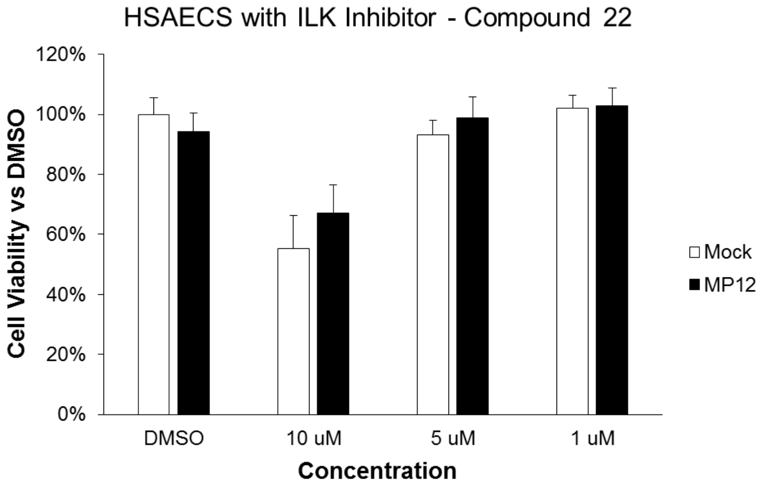

b

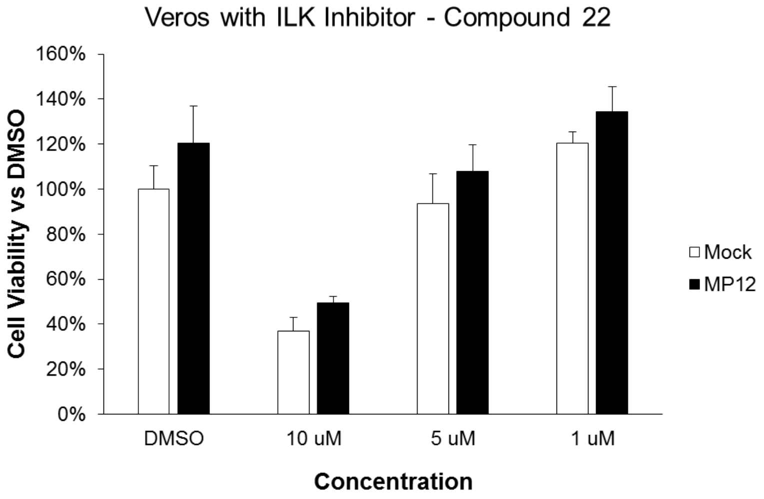

Supplemental Figure 7. Cell viability in cells treated with Compound 22. HSAECs (A) or Veros (B) were pretreated with the indicated concentrations of the ILK inhibitor, Compound 22, for one hour. Following pretreatment, cells were infected with MP12 for one hour, washed, and then complete media (containing no inhibitor) was added back to the cells. At 24 hpi, cells were analyzed for cell viability using Promega's CellTiter Glo reagent according to manufacturer's protocol.

a. Fig 5A

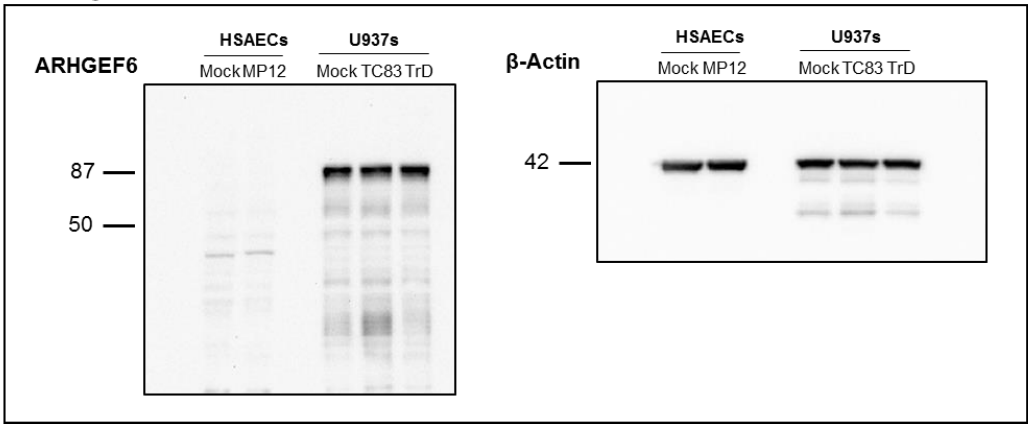

b. Fig 5B

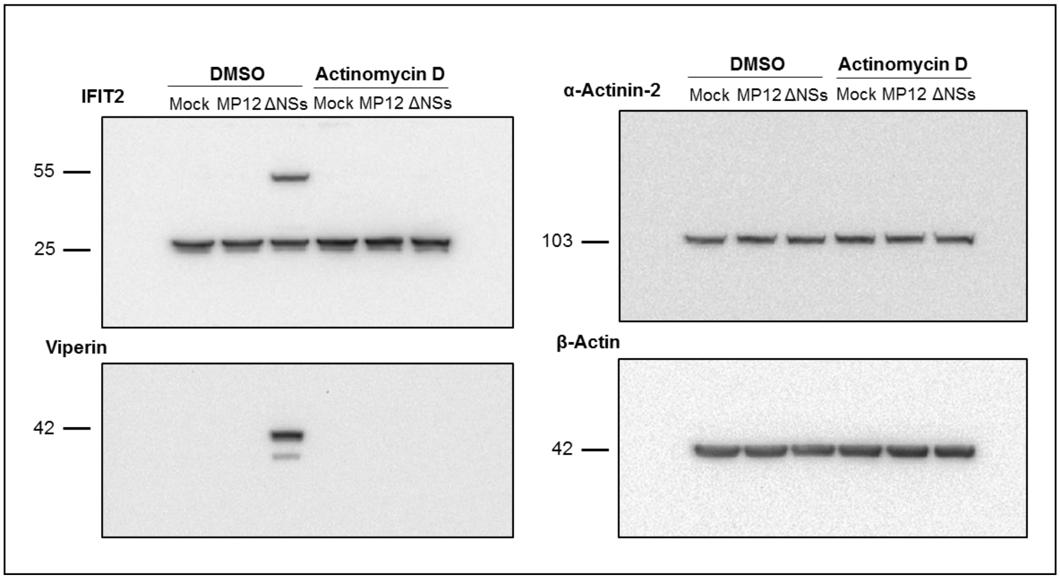

c. Fig 6A

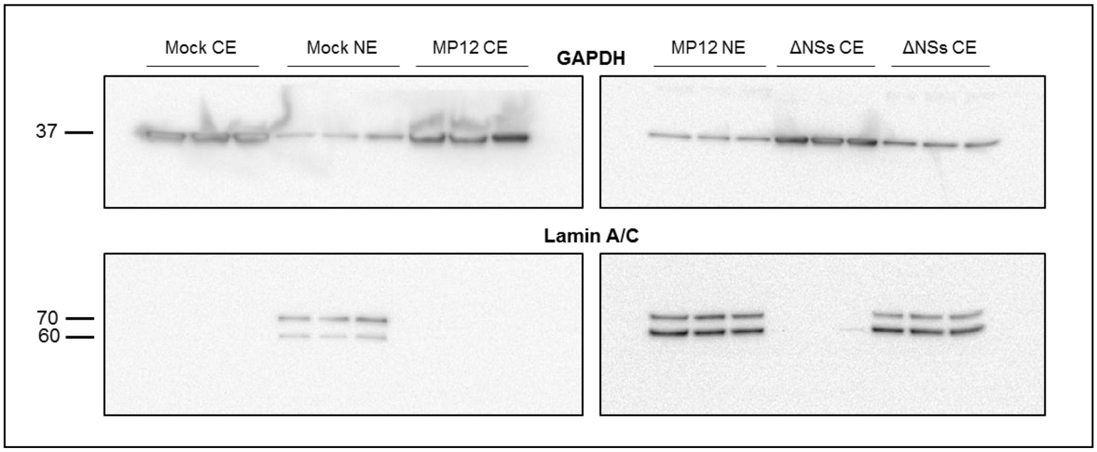

Supplemental Figure 8. Uncropped, original western blots for all experiments.

d. Fig 7A

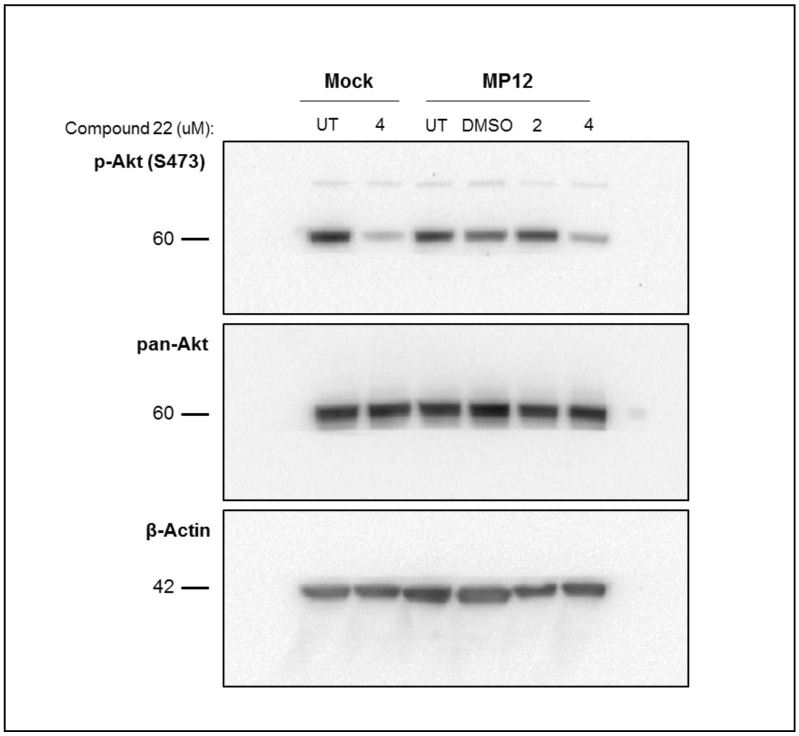

Supplemental Figure 8 (cont'd). Uncropped, original western blots for all experiments.
